# Interpretable side-aware kinematic-sEMG gait-state representations relevant to adaptive neurorobotic assistance after stroke: a public-dataset study

**DOI:** 10.3389/fnbot.2026.1863916

**Published:** 2026-05-25

**Authors:** Rocco Salvatore Calabrò, Andrea Calderone, Alessio Baricich, Andrea Santamato, Francesca Antonia Arcadi, Alessandro Marco De Nunzio, Angelo Quartarone

**Affiliations:** 1 IRCCS Centro Neurolesi Bonino Pulejo, Messina, Italy; 2 Department of Biomedical Sciences, Humanitas University, Milan, Italy; 3 IRCCS Humanitas Research Hospital, Milan, Italy; 4 Physical Medicine and Rehabilitative Unit-Riuniti Hospital, University of Foggia, Foggia, Italy; 5 Department of Research and Development, LUNEX International University of Health, Differdange, Luxembourg; 6 Luxembourg Health and Sport Sciences Research Institute ASBL, Differdange, Luxembourg

**Keywords:** adaptive gait assistance, gait kinematics, latent state discovery, model interpretability, multimodal representation, neurorobotics, post-stroke gait, surface electromyography

## Abstract

**Background:**

Adaptive lower-limb neurorobotics requires gaitd-state representations that preserve locomotor structure without reducing post-stroke walking to a single asymmetry score or opaque latent embedding. Because post-stroke gait is multimodal and side dependent, transparent side-aware representations may better support future adaptive-assistance design than modality-isolated summaries.

**Methods:**

This secondary analysis used a public multimodal gait dataset comprising 138 able-bodied adults and 50 adults with stroke. The analytic space was restricted to 11 waveform domains shared across public exports: four sagittal kinematic waveforms and seven repository-normalized surface electromyography waveforms, each represented by 1,001 time-normalized points. Stroke waveforms were organized into paretic, non-paretic, bilateral-mean, and side-difference views, with side difference defined as paretic minus non-paretic. Domain-view functional principal component analysis retained 90% cumulative variance, capped at three components per block; family-level reduction retained 90% variance, capped at eight components. Candidate Ward hierarchical and K-means solutions from two to five states were screened in kinematics-only, sEMG-only, fused, paretic-only, and erector-spinae-excluded spaces.

**Results:**

The retained fused side-aware solution organized the strict complete-case stroke cohort (*n =* 43) into three states: State 1 (*n =* 12), State 2 (*n =* 18), and State 3 (*n =* 13). The strongest fused two-state K-means comparator showed higher compactness and resampling stability than the retained three-state solution [silhouette 0.189; bootstrap adjusted Rand index (ARI) 0.876 versus silhouette 0.155; bootstrap ARI 0.633]. However, the three-state solution was retained as a representation-level choice because it avoided trivial micro-clusters, preserved explicit multimodal side-aware structure, and enabled clearer waveform-level interpretation. Sensitivity analyses showed identical assignments after erector-spinae exclusion (ARI = 1.000), partial concordance under robust scaling (ARI = 0.785), and material reassignment when the block cap was reduced to two components (ARI = 0.335). The strongest domain contributors were ankle angle (1.000), vastus lateralis sEMG (0.898), knee angle (0.866), gastrocnemius sEMG (0.851), and tibialis anterior sEMG (0.840).

**Conclusion:**

Public waveform exports supported an internally interpretable, side-aware multimodal representation of post-stroke gait relevant to neurorobotic state-representation design. This contribution remains exploratory and representational, not clinical, interventional, real-time, or controller-validating; for future studies, it should be interpreted as a hypothesis-generating framework.

## 1 Introduction

Post-stroke walking is not a single impairment with a single technical solution. It is a heterogeneous motor behavior that emerges from interacting deficits in propulsion, coordination, balance control, timing, muscle recruitment, and sensorimotor adaptation. This complexity helps explain why gait has become a prominent target for artificial intelligence in neurorehabilitation. Contemporary reviews show that stroke rehabilitation is now one of the main clinical settings in which artificial intelligence is being used to process movement data, and gait is among the most common application domains because locomotion generates rich, high-dimensional signals that are difficult to summarize through visual inspection alone (Senadheera et al., 2024; Sumner et al., 2023). At the same time, recent commentaries in neurorehabilitation have emphasized that technological measurement will have limited value unless it supports decisions that matter for therapy, monitoring, and adaptive assistance rather than producing isolated analytic novelty (Cain and Leech, 2025).

This translational pressure exposes an important limitation of conventional post-stroke gait assessment. Even when sophisticated laboratory data are available, interpretation is often collapsed into gait speed, endurance, or broad observational scores. Those outcomes remain clinically useful, yet synthesis studies of post-stroke kinematics show that they do not fully represent how walking is organized across joints and phases of the gait cycle (Li et al., 2025). The same concern applies to asymmetry. A recent meta-analysis demonstrated that temporal and spatial asymmetry are related to partly distinct stroke-associated factors and should not be treated as interchangeable manifestations of one underlying deficit (Xu et al., 2025). Earlier work in community-ambulating stroke survivors reached a compatible conclusion, showing that substantial asymmetry may persist despite acceptable functional walking capacity (Patterson et al., 2008). For technologies that aim to interact with the user during walking, these hidden differences are not trivial. They indicate that similar overall performance can arise from different neuromechanical strategies.

That issue becomes central in neurorobotics. Lower-limb rehabilitation robots, powered exoskeletons, and related wearable systems are increasingly expected to adapt their assistance to the user rather than impose a fixed trajectory. Reviews in recent neurorobotics and stroke-robotics literature consistently describe the same challenge: effective assistance depends on estimating patient state, movement intention, and changing support needs from multimodal signals instead of relying exclusively on preprogrammed schedules (Su et al., 2023; Hobbs and Artemiadis, 2020). Experimental studies in stroke rehabilitation have likewise shown that exoskeleton-assisted training can alter neuromuscular organization and that individualized robotic gait guidance may influence energetic and motor behavior in ways that generic trajectories do not fully capture (Longatelli et al., 2021; Guo et al., 2022). In other words, neurorobotic gait support needs internal representations of the walking pattern that are both data-rich and behaviorally meaningful.

The literature on human-machine interfacing offers several clues about what those representations may require. Gait phase classification in rehabilitation exoskeletons has benefited from combining multiple information streams, including surface electromyography, joint kinematics, and plantar or interaction signals, rather than depending on one modality alone (Zhang et al., 2021). Related work has shown that surface electromyography can anticipate limb motion and improve lower-limb kinematic prediction when it is fused with phase information in exoskeleton-oriented decoding pipelines (Wei et al., 2021). More recent reviews of neuro-motor controlled wearable augmentations have extended that argument beyond single devices, emphasizing that embodied assistive systems increasingly depend on multimodal sensing, state estimation, and user-specific control variables that remain interpretable to both engineers and clinicians (Alsuradi et al., 2024). These developments make stroke gait analysis relevant to neurorobotics not only as an assessment problem, but also as a representation problem.

Model interpretability, rather than a claim of advanced *post-hoc* explanation methodology, is central to this representation problem. In rehabilitation robotics, transparent signal-to-state mapping is important for human oversight, controller auditability, and clinically credible adaptation. Stakeholder-driven reviews of interpretable artificial intelligence in medicine have linked transparency to trust, deployment, and effective human-AI team performance (Subramanian et al., 2024), and broader healthcare perspectives have emphasized that computational systems influencing treatment or device behavior require understandable outputs (Amann et al., 2020). For gait-oriented neurorobotics, black-box discrimination between stroke and non-stroke walking is therefore of limited value. What matters more is whether multimodal waveforms can be transformed into state representations that show which locomotor domains structure the state space, how side-dependent organization differs across individuals, and which signals may be worth estimating in future adaptive-assistance systems.

Current rehabilitation-robotics research already points in that direction. Assistance-level quantification and human-robot interaction space reshaping have been proposed to link electromyographic and kinematic information to assist-as-needed behavior (Li et al., 2023). Reinforcement-learning-based continuous mode adaptation has been explored to modify robotic operation according to changing user participation (Yang et al., 2022). Methodological work on human-exoskeleton interaction has also highlighted the need for interpretable metrics that connect muscle activation, joint behavior, and assistance effects rather than treating them as disconnected outputs (Fanti et al., 2022). Yet these studies also make clear that controller-oriented metrics cannot be imported directly from generic gait analysis. They require structured representations that are sensitive to side-dependent pathology, preserve waveform organization, and remain stable enough to support future decision layers.

Post-stroke gait provides a strong test case for that problem because asymmetry and compensation are distributed across the body rather than confined to one joint or one signal family. Recent work has shown that modifying propulsion asymmetry within a session does not automatically normalize the broader gait pattern (Kettlety et al., 2025). Trunk adaptations can also remain deeply embedded in hemiplegic walking, indicating that post-stroke locomotion is organized through coupled lower-limb and axial strategies rather than isolated segmental deficits (Van Criekinge et al., 2017). The present study was designed within that conceptual space. Using only the public spreadsheet exports and repository documentation available for the Van Criekinge dataset, we developed an interpretable kinematic-sEMG framework for subject-level gait-state representation after stroke. The aim was to derive side-aware multimodal waveform representations, identify latent state structure within the stroke cohort, and determine which domains contributed most strongly to state separation, while explicitly limiting the claims to representation design rather than clinical diagnosis, online state estimation, or controller validation.

## 2 Materials and methods

### 2.1 Study design

This study was designed as a secondary analysis of a deidentified, publicly available gait dataset, with reporting aligned to established principles for observational research and contemporary methodological guidance for machine learning applications in health data science (von Elm et al., 2007; Sengupta et al., 2020). The primary objective was not supervised diagnosis, clinical outcome prediction, clinical subtyping, online state estimation, or validation of a real-time robotic controller. The purpose was instead to develop an interpretable subject-level representation of post-stroke gait that could inform future neurorobotic applications requiring adaptive, patient-specific state estimation. This distinction shaped the design from the outset. The analytic workflow was intentionally limited to public spreadsheet exports and repository documentation so that every transformation, feature-construction step, and representation comparison could be audited from the public waveform layer without reprocessing raw C3D files, hidden stride arrays, proprietary source-processing routines, or non-public metadata.

The workflow followed four successive stages. First, the signal space was restricted to waveform domains genuinely shared across the able-bodied and stroke spreadsheet exports. Second, those waveforms were transformed into side-aware subject-level representations intended to retain locomotor meaning while reducing dimensionality. Third, latent structure was explored within the stroke cohort using unsupervised learning rather than pre-existing gait classes or a stroke-versus-control classifier. Fourth, model-interpretability procedures were applied to determine which kinematic and sEMG domains most strongly shaped the resulting state structure. This sequence was chosen to keep the study aligned with neurorobotic questions of state representation, adaptive-assistance design, and robustness screening within a public multimodal dataset.

### 2.2 Public multimodal dataset and repository files

The source data came from the public multimodal gait resource described by Van Criekinge and colleagues, which includes 138 able-bodied adults and 50 adults with stroke assessed using harmonized overground gait-laboratory procedures (Van Criekinge et al., 2023). The full repository contains raw C3D files, post-processed MATLAB structures, spreadsheet exports, documentation files, example code, and time-normalized figures. The present analysis deliberately restricted itself to the public spreadsheet exports and the repository documentation. This choice was methodological rather than merely practical. It ensured that the manuscript depended only on files that could be inspected and reproduced directly by secondary analysts, and it prevented the inference pathway from being influenced by source structures that were not part of the spreadsheet-based public analytic layer.

The repository descriptor indicates that the original acquisition environment combined full-body marker-based gait analysis, synchronized force-plate measurements, and surface electromyography within a conventional clinical gait-laboratory workflow (Van Criekinge et al., 2023; Armand et al., 2024). For the purposes of the present manuscript, those upstream procedures were treated as the context from which the public waveforms emerged. The study did not re-estimate kinematics from markers, reselect gait events, or reinterpret raw force-plate recordings. Instead, it treated the public waveform exports as curated functional summaries that could support a representation-learning question relevant to adaptive neurorobotic systems.

### 2.3 Participants and shared waveform space

All participants present in the public spreadsheet exports were considered eligible for the relevant stage of analysis. The able-bodied cohort was used to define the shared public waveform space and to provide descriptive context for typical waveform organization, but it was not the target of latent gait-state discovery. The stroke cohort was the target population for latent gait-state modelling. The public data descriptor reports that the able-bodied cohort ranged from 21 to 86 years of age, whereas the stroke cohort ranged from 19 to 85 years and was evaluated in the subacute period after first-ever stroke (Van Criekinge et al., 2023). Because the spreadsheet exports used as direct analytic input did not provide a full participant-level clinical metadata table, no additional subgroup stratification by lesion side, stroke subtype, or impairment severity was prespecified.

Inspection of the spreadsheet exports showed that the able-bodied workbook contained one participant-level waveform per variable, whereas the stroke workbook contained separate paretic and non-paretic waveforms. The able-bodied public spreadsheet layer therefore could not be used to construct a homologous left–right or paretic/non-paretic asymmetry representation. The able-bodied cohort was consequently used for shared-domain eligibility and descriptive reference context rather than for direct side-aware state discovery. The able-bodied workbook included additional kinetics and ground-reaction-force variables that were not exported in the stroke workbook. To preserve strict comparability and avoid state definitions driven by variables unavailable in the stroke cohort, the shared analytic space was limited to eleven waveform domains: four sagittal kinematic waveforms, namely ankle, knee, hip, and pelvis angles, and seven normalized sEMG waveforms, namely gastrocnemius, rectus femoris, vastus lateralis, biceps femoris, semitendinosus, tibialis anterior, and erector spinae. Repository documentation specifies that normalized sEMG amplitudes were normalized to the maximum value of each individual’s muscle during the gait cycle. Every waveform spanned the gait cycle with 1,001 equidistant samples, which allowed the gait cycle to be treated as a functional object rather than as a small set of discrete events. The participant-level waveform, not the stride and not the trial, was the fundamental analytic unit. At the raw spreadsheet-export level, all four paired kinematic domains were complete in 50/50 stroke participants. Bilateral normalized lower-limb sEMG completeness was lower and channel dependent: gastrocnemius, rectus femoris, vastus lateralis, biceps femoris, semitendinosus, and tibialis anterior were complete in 43/50 paired cases, whereas erector spinae was complete in 46/50 paired cases. Accordingly, all 50 stroke participants contributed to repository-level availability reporting, whereas the strict fused complete-case cohort used for retained state discovery was limited to the 43 participants with paired lower-limb normalized sEMG across the shared domains; the seven excluded participants are documented in the subject-level inclusion materials.

### 2.4 Source processing context and preprocessing logic

Although no raw files were reprocessed, the source-processing context remained important because it defines the credibility of the public waveforms. The public descriptor and repository documentation indicate that the dataset arose from a standard marker-based clinical gait-analysis workflow supported by synchronized electromyography acquisition and subsequent time normalization (Van Criekinge et al., 2023; Armand et al., 2024). Established recommendations for surface electromyography sensor placement and signal preparation are also relevant when interpreting the exported electromyography channels (Hermens et al., 2000). These upstream considerations justify treating the spreadsheet waveforms as biomechanically coherent summaries rather than minimally processed sensor streams.

Preprocessing within the present study was intentionally conservative. All exported columns were imported exactly as distributed. Numeric integrity was checked domain by domain, and each waveform was screened for missing samples, constant-value artifacts, or dispersion patterns suggesting import problems rather than genuine locomotor behavior. No additional low-pass filtering, gait-cycle realignment, derivative expansion, phase re-segmentation, raw EMG re-normalization, or imputation of missing sEMG waveforms was performed. These operations could materially alter waveform morphology and would be difficult to justify without returning to the full source structures. Missing paired lower-limb sEMG was therefore handled through strict complete-case analysis for fused state discovery, with the excluded participants retained in the availability and assignment documentation. The guiding principle was that the public waveform itself should remain the final functional object for analysis. Where point wise inspection of waveforms informed descriptive review, the gait cycle was treated as a continuous one-dimensional object rather than as a sequence of unrelated time points, consistent with accepted treatment of biomechanical trajectories (Pataky, 2012).

### 2.5 State-oriented representation and feature extraction

The main representational challenge was dimensionality combined with side dependence. Direct point-by-point concatenation of all stroke waveforms would have produced a feature vector far larger than the cohort size and would have obscured the distinction between unilateral abnormality and interlimb organization. To preserve neurorobotic relevance, each domain was represented through multiple complementary views. For every kinematic and sEMG domain, four subject-level waveform views were constructed: the paretic waveform, the non-paretic waveform, the bilateral mean waveform, and the side-difference waveform. The side-difference sign was defined as paretic minus non-paretic throughout the analysis, so positive or negative values retained directional asymmetry rather than being converted into unsigned asymmetry magnitude. The first two views preserved absolute side behavior. The bilateral mean view captured global locomotor organization. The side-difference view explicitly encoded interlimb imbalance, which is relevant when future assistive systems may need to adapt support according to asymmetry rather than according to unilateral deficit alone.

Before dimensionality reduction, each domain-view waveform was centered and scaled at the point wise level within cohort so that kinematics and normalized sEMG could be fused without one signal family dominating the reduced space. This within-cohort point wise standardization was used as the primary scaling strategy in the retained representation, while robust scaling was reserved for sensitivity analysis. Dimensionality reduction was performed within each domain-view combination rather than across all waveforms simultaneously. Functional principal component analysis was selected because it can compress high-dimensional curves into a limited set of coefficients while retaining a direct link to waveform shape. Components were retained to 90% cumulative variance subject to a cap of three components per domain-view block. The assembled family-level feature spaces were then reduced again to 90% cumulative variance subject to a cap of eight family components before clustering. Component-retention sensitivity was tested by lowering the block cap to two components and increasing it to four components.

In parallel with the functional representation, a lower-complexity descriptor set was constructed for interpretability support. These scalar summaries included waveform level, peak behavior, integrated magnitude, timing-related summaries, and side-difference burden. They were not intended to replace the functional representation in the primary modelling stage. Their role was to facilitate interpretation of the final latent structure and to provide a transparent bridge between high-dimensional coefficients and recognizable gait behavior. Three modality-specific representation families were prespecified: a kinematics-only space, an sEMG-only space, and a fused kinematic-sEMG space. Additional sensitivity families removed erector spinae information or collapsed the representation to a paretic-only view. These representation families were compared to determine whether multimodal side-aware fusion yielded more behaviorally coherent latent gait states than simpler modality-specific or reduced-view alternatives. Raw public-export completeness across all 11 shared domains was documented separately from the fitted fused solution. The final fused state space was estimated in the strict complete-case stroke cohort (*n =* 43), whereas the full 50-participant stroke workbook was preserved for repository-level availability reporting and descriptive completeness summaries.

### 2.6 Latent gait-state discovery and neurorobotic relevance

Latent structure was explored exclusively within the stroke cohort. Candidate state solutions were generated through unsupervised clustering performed on the reduced feature spaces. The candidate grid screened Ward hierarchical clustering and K-means clustering across two-, three-, four-, and five-state solutions within each representation family. Because the cohort was modest and because neurorobotic relevance depends more on coherent state structure than on maximal granularity, the search was restricted to low-cardinality partitions that could plausibly support waveform-level interpretation. Solutions were evaluated using compactness and separation indices, including silhouette, Calinski-Harabasz, and Davies-Bouldin metrics, together with graphical embedding inspection, repeated 80% subsampling with adjusted Rand index (ARI) summaries, minimum cluster size, and back-projected waveform interpretability.

This approach was grounded in earlier work showing that cluster analysis can reveal stroke gait heterogeneity not apparent from cohort averages alone (Mulroy et al., 2003). The present study extends that logic by using multimodal waveforms and by framing the clusters as candidate representation states rather than externally validated clinical classes. A state solution was considered acceptable only if it avoided trivial micro-clusters, remained sufficiently stable under mild perturbation, preserved explicit side-aware structure when that was the target family, and could be interpreted through identifiable domain contributions and recognizable waveform differences. When multiple candidate solutions satisfied these conditions, retention favored the lowest-cardinality multimodal solution that preserved side-aware kinematic-sEMG fusion and supported waveform-level interpretation. These criteria were operational selection criteria for this secondary analysis, not pre-registered clinical classification criteria.

The neurorobotic interpretation of these states was deliberately restrained. The present study does not claim that the latent states are ready to drive a controller directly. Rather, it treats them as representation-level candidates for future decision layers in systems that may adapt assistance according to patient-specific locomotor organization. This distinction is important because neurorobotic relevance depends on representation layers that can eventually support intelligent embodied assistance, whereas generic sensor descriptions or unvalidated control claims would overstate what a public-dataset analysis can deliver. The present work therefore stops at the level of state representation, separability, interpretability, and robustness screening.

### 2.7 Model interpretability, robustness, reproducibility, and ethics

Model interpretability was integrated into the workflow rather than added after clustering. Once a candidate state solution had been selected, centroid structures were back-projected into waveform space so that the original kinematic and sEMG domains could be inspected in their native locomotor form. This step was essential because latent coefficients have limited translational value unless they can be translated back into recognizable gait signatures. Domain-level contribution was quantified directly in standardized waveform space by aggregating the between-state separation fraction across the four side-aware views of each domain and normalizing the resulting score to the highest-contributing domain. These values were used as transparent interpretive aids rather than as causal importance estimates or as advanced *post-hoc* explanation outputs. Recent reviews of model interpretability in gait analysis support the need to connect computational outputs to biomechanical signal logic rather than relying on performance metrics alone (Xiang et al., 2025).

Several robustness analyses were prespecified. First, the full workflow was repeated with robust scaling instead of standard z-scaling. Second, the cap on retained functional components was varied to determine whether the latent structure depended on a narrow dimensionality choice. Third, the representation families were analysed separately and jointly to determine whether multimodal fusion contributed coherent state information or merely added variance. Fourth, the full analysis was repeated after excluding the erector spinae channel because axial electromyography may provide valuable coordination information in some stroke participants but may also inflate representation variance unrelated to lower-limb assistance logic. Fifth, side-aware representations were compared with paretic-only representations to test whether explicit asymmetry encoding improved state consistency. These robustness checks were chosen because waveform similarity and symmetry metrics have long been recognized as sensitive but method-dependent descriptors in clinical gait analysis (Iosa et al., 2014; Patterson et al., 2010).

The workflow was designed to be transparent and reproducible at the documented pipeline level from the public repository files. At manuscript level, reproducibility refers to deterministic reconstruction of the representation workflow from the public waveform layer, whereas exact stochastic regeneration of the same state assignments and permutation-style interpretability summaries requires the executable implementation metadata. The archived materials document the public inputs, waveform structure, shared-domain restriction, dimensionality caps, candidate clustering screen, and 80% subsampling design. Exact random seed values, exact resampling counts, and exact permutation counts were not fully enumerated in the manuscript-level archive and should accompany any executable code release. No proprietary preprocessing environment was required beyond standard statistical tools capable of reading spreadsheets and applying deterministic feature-construction steps. The study used only deidentified public data, and no new participant contact, intervention, or data collection occurred. Ethical approval and informed consent procedures for the original data collection are described in the public dataset report (Van Criekinge et al., 2023).

**Figure 1:**
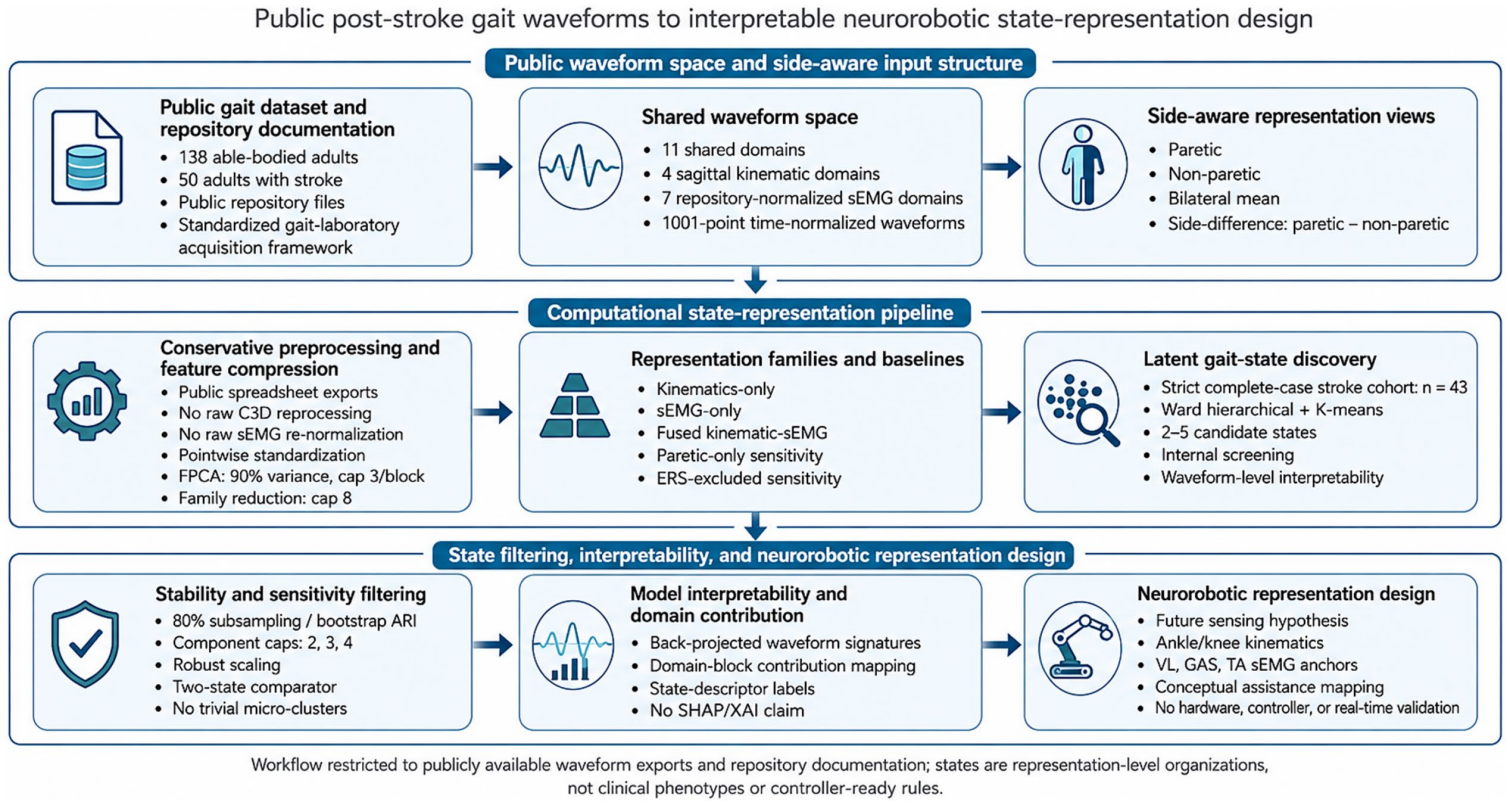
Computational pipeline for side-aware multimodal gait-state discovery. The workflow summarizes the transition from publicly available waveform exports and repository documentation to side-aware representation construction, latent gait-state discovery, model interpretability, and neurorobotic representation-design relevance. The pipeline is restricted to representation-level analysis and does not imply clinical phenotyping, hardware validation, real-time state estimation, or controller validation.

**Figure 2:**
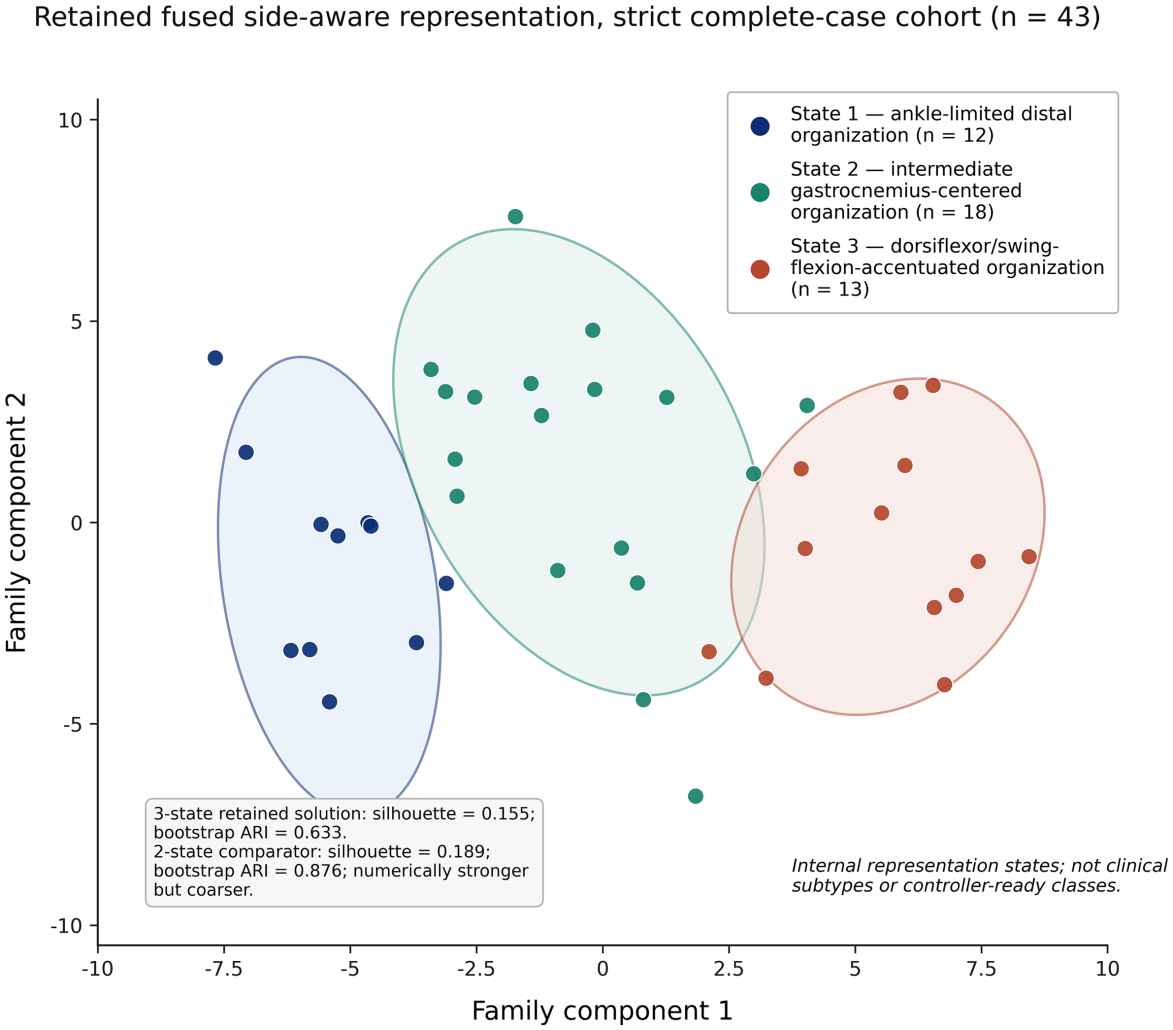
Low-dimensional organization of side-aware multimodal gait states in the stroke cohort. The plot shows the retained fused side-aware three-state solution in the strict complete-case stroke cohort. Coordinates correspond to the first two family-level components reported in Supplementary material 7. Ellipses are descriptive visual summaries of internal organization and do not represent externally validated clinical classes.

**Figure 3:**
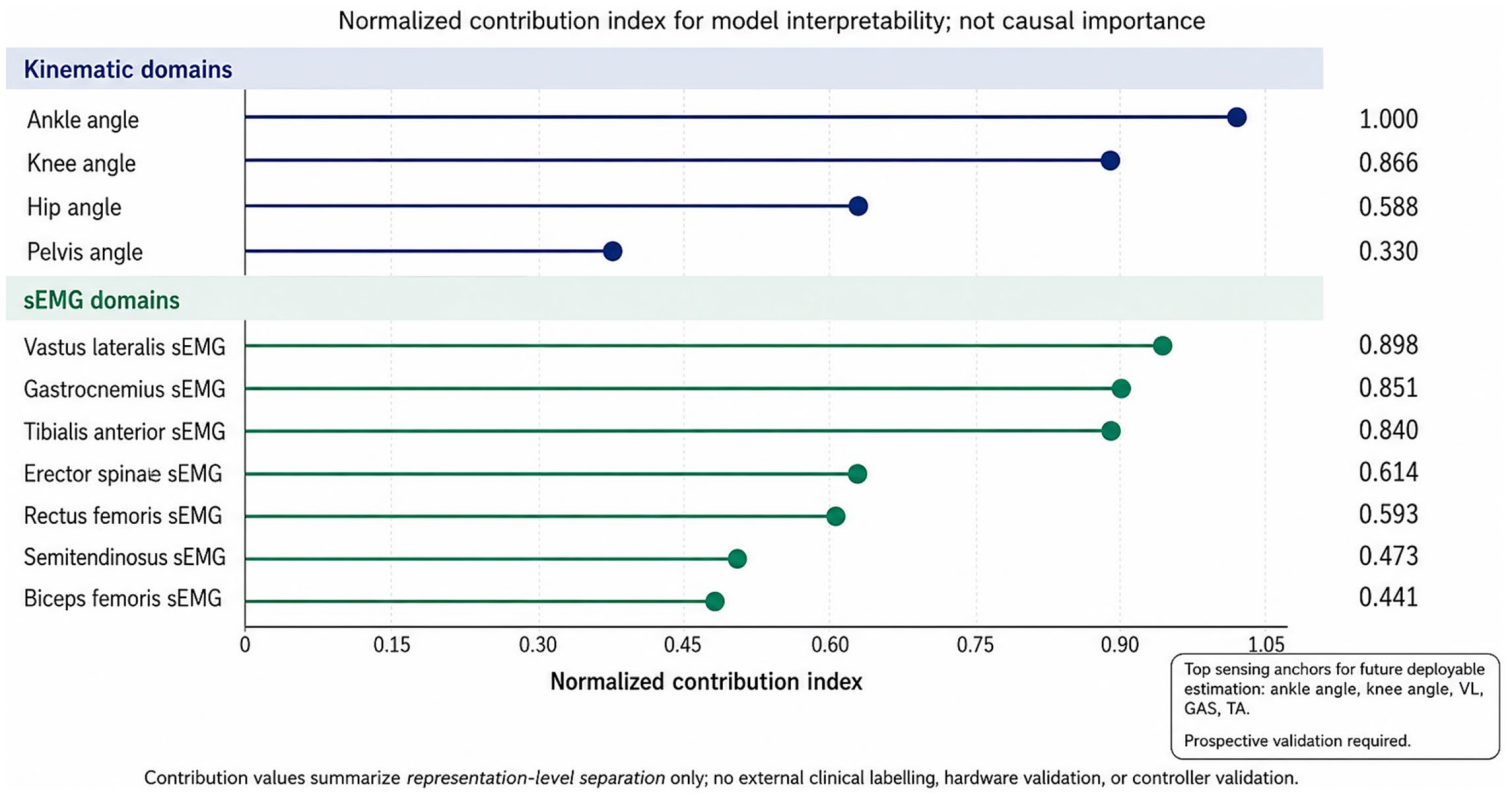
Domain-level contribution to retained side-aware multimodal gait-state separation. The lollipop chart shows the normalized domain-level contribution index for the retained fused side-aware solution. Values summarize representation-level separation only and should not be interpreted as causal importance, external clinical labelling, hardware validation, or controller validation.

**Figure 4:**
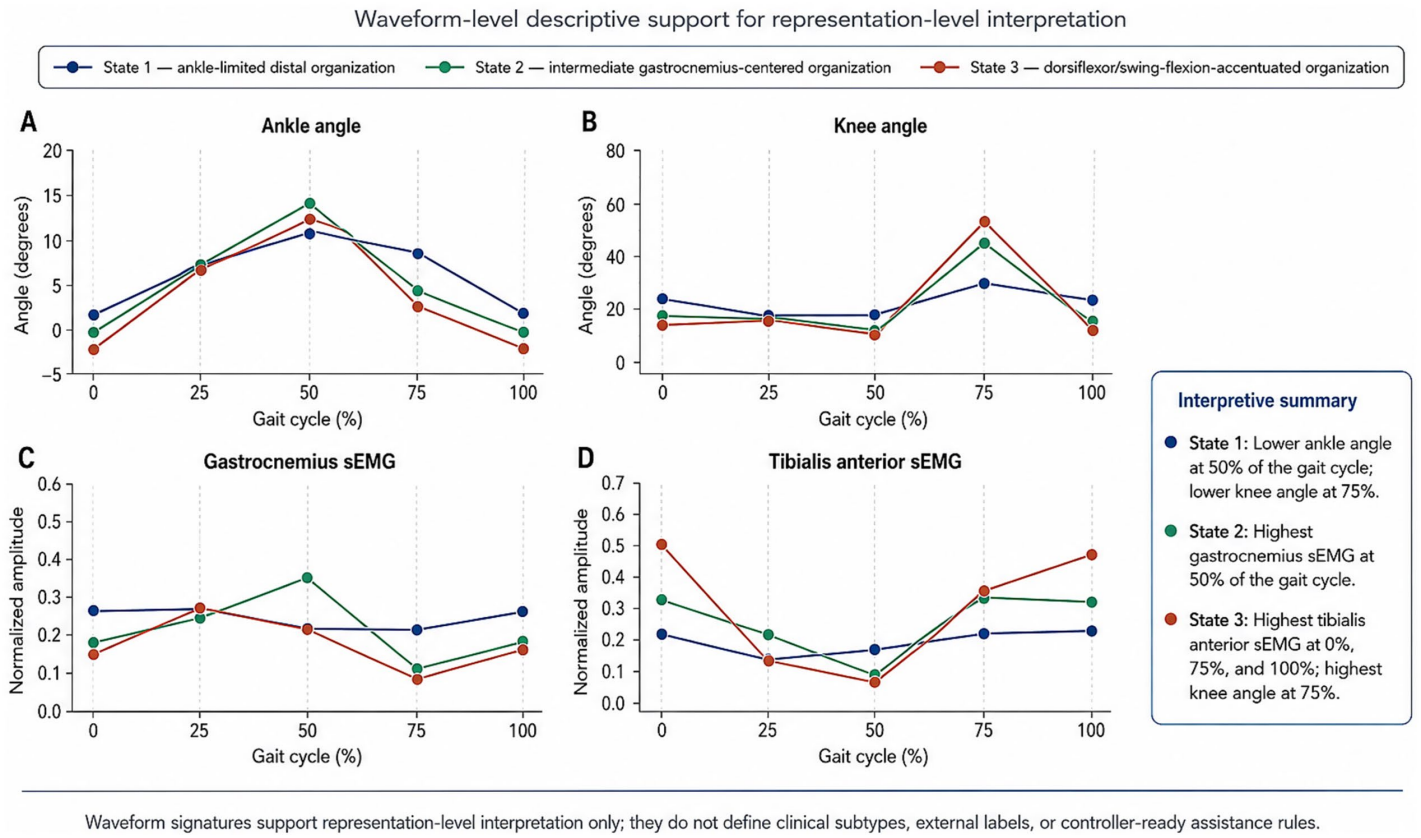
Compact biomechanical signatures of the retained side-aware gait states. State-wise descriptive summaries are shown for representative waveform domains, including ankle angle, knee angle, gastrocnemius sEMG, and tibialis anterior sEMG, at 0, 25, 50, 75, and 100% of the gait cycle. The panels support representation-level interpretation of the retained states and do not define clinical subtypes, external labels, or controller-ready assistance rules.

During manuscript preparation, ChatGPT was used only to assist with graphical layout refinement and visual formatting of Figures 1–4. The tool was not used to generate, modify, or interpret numerical data, perform analyses, create latent-state assignments, derive statistical outputs, write or select references, or make scientific decisions. All figure content, labels, numerical values, and data-to-visual mappings were checked by the authors against the underlying analyses and manuscript results. The authors take full responsibility for the accuracy, integrity, and final content of all figures.

## 3 Results

### 3.1 Final analytic space and retained representation logic

At the repository level, the analysis started from 138 able-bodied adults and 50 adults with stroke. The public waveform layer was restricted to the signal domains genuinely shared across both exports so that downstream comparisons remained anchored to the same observable space. This restriction yielded 11 domains, comprising ankle, knee, hip, and pelvis sagittal-angle waveforms together with seven normalized sEMG waveforms from gastrocnemius, rectus femoris, vastus lateralis, biceps femoris, semitendinosus, tibialis anterior, and erector spinae. Every retained waveform was represented as a 1,001-point time-normalized trajectory over the gait cycle. Paired kinematic coverage was complete in all 50 stroke participants. In contrast, paired lower-limb normalized sEMG completeness was 43/50 for gastrocnemius, rectus femoris, vastus lateralis, biceps femoris, semitendinosus, and tibialis anterior, and paired erector spinae completeness was 46/50. The seven participants with missing paired lower-limb normalized sEMG were excluded only from strict fused complete-case state discovery and remained documented in the subject-level availability table. The dataset structure, analytic cohort logic, and shared-domain restriction are summarized in Table 1.

**Table 1:** Public dataset structure, analytic cohort, and side-aware shared waveform space.

Component	Able-bodied repository layer	Stroke repository layer	Role in the present study	Analysis-ready note
Public cohort size	138 able-bodied adults	50 adults with stroke	Descriptive context cohort for shared-domain eligibility and typical waveform organization versus target cohort for latent gait-state discovery	Directly supported by the public repository descriptor and spreadsheet workbooks
Age range and stage	21–86 years	19–85 years; subacute after first-ever stroke	Repository-level descriptive context only; not used for subgroup stratification in the spreadsheet-based workflow	The direct spreadsheet layer does not provide a complete clinical metadata table for subgroup modelling
Workbook structure	ReadMe sheet plus 138 subject tabs	ReadMe sheet plus 50 subject tabs	Subject-level spreadsheet export used as the direct analytic layer	One participant-level waveform block per worksheet
Analytic unit	One subject-level averaged waveform per exported variable	One subject-level paretic waveform and one subject-level non-paretic waveform per exported variable	The participant-level waveform, rather than the stride or trial, is the fundamental analytic unit	All exported waveforms span the full gait cycle
Waveform length	1,001 equidistant samples per variable	1,001 equidistant samples per variable	Supports treatment of gait trajectories as functional observations	Repository documentation states equal normalization across the gait cycle
Shared sagittal kinematic block	Ankle, knee, hip, and pelvis angles	Paretic and non-paretic ankle, knee, hip, and pelvis angles	Four-domain kinematic family entering side-aware state representation	All four kinematic domains are complete in the stroke cohort
Shared normalized sEMG block	GAS, RF, VL, BF, ST, TA, and ERS channels	Paretic and non-paretic GAS, RF, VL, BF, ST, TA, and ERS channels	Seven-domain myoelectric family entering side-aware state representation	Channel completeness varies across subjects and is the main source of complete-case reduction
Additional exported variables excluded	Ankle, knee, and hip moments; ankle, knee, and hip powers; three ground-reaction-force components	Not available in the public stroke spreadsheet export	Excluded to prevent latent states from being driven by variables unavailable in the target stroke cohort	This exclusion preserves strict cross-cohort comparability within the public spreadsheet layer
Raw bilateral kinematic completeness	138/138 complete across all four kinematic domains	Paretic 50/50; non-paretic 50/50; paired side-aware 50/50	All kinematic views remain available for the full stroke cohort	Supports 16 kinematic domain-view blocks (4 domains × 4 views)
Raw bilateral normalized sEMG completeness	102/138 complete across all seven normalized sEMG domains	Paretic 43/50; non-paretic 44/50; paired side-aware 43/50	Defines the largest complete-case set for myoelectric-only state discovery	ERS is available in 46 paired cases; the lower-limb EMG channels are complete in 43 paired cases
Strict raw completeness across all 11 shared domains	102/138 complete across all eleven shared domains	Paretic 43/50; non-paretic 44/50; paired side-aware 43/50	Documents the most restrictive raw availability profile across the shared domains; it should not be read as a direct synonym of downstream retained-state membership	The fused family is limited by the lower-limb sEMG completeness profile
Derived side-aware representation views	Native workbook contains one side-averaged waveform per domain	Native workbook contains separate paretic and non-paretic waveforms	Stroke-domain views were expanded into paretic, non-paretic, bilateral mean, and side-difference representations	This yields 44 domain-view combinations from the 11 shared domains

The able-bodied spreadsheet workbook contains one side-averaged waveform per subject and therefore does not provide direct left–right asymmetry exports. Bilateral-mean and side-difference views are derived within the stroke cohort from paired paretic and non-paretic waveforms. sEMG, surface electromyography; GAS, gastrocnemius; RF, rectus femoris; VL, vastus lateralis; BF, biceps femoris; ST, semitendinosus; TA, tibialis anterior; ERS, erector spinae. This table anchors to the public repository layer used directly for analysis. It distinguishes the descriptive role of the able-bodied cohort from the state-discovery role of the stroke cohort, and it clarifies which waveform domains remain eligible after strict restriction to genuinely shared variables. The final column summarizes the availability conditions that govern whether a representation family can be carried forward into side-aware modelling.

The primary workflow treated side dependence as a structural property of the representation rather than as a *post-hoc* descriptor. For every shared stroke domain, four views were carried forward: the paretic waveform, the non-paretic waveform, a bilateral mean, and a side-difference signal defined as paretic minus non-paretic. This design allowed the analysis to encode unilateral abnormality, whole-pattern organization, and directional asymmetry burden within the same feature system. Domain-wise within-cohort pointwise standardization was followed by FPCA compression to 90% cumulative variance with a cap of three components per block, then family-level reduction to 90% cumulative variance with a cap of eight components. The resulting candidate spaces contained 16 side-aware kinematic blocks, 28 side-aware sEMG blocks, and 44 fused multimodal blocks. The full representation framework and feature-construction pipeline are outlined in Table 2.

**Table 2:** State-oriented representation framework and feature-construction pipeline.

Pipeline element	Input layer	Output object	Function in the state-discovery workflow	Constraint or implementation note
Shared-domain restriction	Public spreadsheet exports from the able-bodied and stroke workbooks	Eleven genuinely shared domains	Restricts representation learning to signals available in both cohorts	Prevents state definitions from depending on kinetics or force variables absent from the public stroke export
Subject-level waveform import	1,001-point participant-level trajectories	Domain-wise functional objects	Preserves the repository waveform as the final analytic object	No re-estimation from markers, force plates, or raw EMG source files
Side-aware view construction	Stroke paretic and non-paretic waveforms	Paretic, non-paretic, bilateral mean, and side-difference views	Separates unilateral abnormality, global locomotor organization, and interlimb asymmetry	Four views are constructed per domain when paired stroke data are available
Within-cohort pointwise centering and scaling	Stroke domain-view waveforms within each representation family	Standardized domain-view inputs	Places kinematics and sEMG on a comparable footing before multimodal fusion	Robust-scaling variants are reserved for sensitivity analysis
Domain-view compression	Standardized 1,001-point curves	Reduced coefficient sets	Controls dimensionality while preserving waveform shape information	FPCA retained 90% cumulative variance subject to a cap of three components per domain-view block; two- and four-component caps were tested as sensitivity analyses
Interpretability support layer	Original curves and reduced coefficients	Lower-complexity scalar descriptors	Provides an auditable bridge between latent coordinates and recognizable gait behavior	Descriptor families include waveform level, peak behavior, integrated magnitude, timing-related summaries, and side-difference burden
Representation families	Reduced coefficients and support descriptors	Kinematics-only, sEMG-only, and fused state spaces	Tests whether multimodal fusion yields more stable and behaviorally coherent gait states than single-modality inputs	The kinematic family contains 16 domain-view blocks, the sEMG family 28, and the fused family 44; assembled family spaces were reduced to 90% cumulative variance with a cap of eight components
Latent-state discovery and interpretation	Representation-family feature spaces	Candidate gait-state solutions with back-projected signatures and domain-block importance maps	Links separability to interpretable signal structure relevant to future neurorobotic state estimation	Ward hierarchical and K-means clustering were screened across two to five states; the workflow stops at representation, separability, and interpretation rather than controller validation

This table summarizes the transformation pathway from public repository waveforms to subject-level feature spaces suitable for latent gait-state discovery. Each row corresponds to a processing stage that preserves interpretability while progressively reducing dimensionality. Interpretability-support steps are listed explicitly because neurorobotic relevance depends on transparent signal-to-state mapping rather than on opaque compression alone. The table reports the operational workflow used in the revised analysis, including explicit dimensionality-reduction thresholds and clustering settings. sEMG, surface electromyography; FPCA, functional principal component analysis.

Latent gait-state discovery was screened across kinematics-only, sEMG-only, and fused side-aware families, together with paretic-only and erector-spinae-excluded sensitivity families. Candidate Ward hierarchical and K-means solutions were evaluated from two to five states. Candidate solutions were not retained when they fragmented into trivial micro-clusters, reassigned participants unstably under mild perturbation, or produced signatures that could not be traced back to recognizable signal structure. Within this framework, the fused side-aware family was carried forward as the primary representation because it preserved both movement-trajectory and myoelectric organization while remaining interpretable at the level of individual signal domains. The candidate families and internal selection logic are summarized in Table 3.

**Table 3:** Candidate latent gait-state solutions and internal selection criteria.

Representation family	Waveform coverage	State-number search	Primary internal screening criteria	Automatic rejection triggers	Role in retained-solution selection
Kinematics-only side-aware family	Four sagittal kinematic domains expressed through four views each (16 domain-view blocks)	Ward hierarchical and K-means solutions screened across 2, 3, 4, and 5 states	Compactness, separation, embedding coherence, resampling stability, and interpretability	Unstable reassignment under mild perturbation, trivial micro-clusters, or waveform signatures that cannot be interpreted biomechanically	Single-modality benchmark for movement-trajectory-driven state structure
sEMG-only side-aware family	Seven normalized sEMG domains expressed through four views each (28 domain-view blocks)	Ward hierarchical and K-means solutions screened across 2, 3, 4, and 5 states	Compactness, separation, embedding coherence, resampling stability, and interpretability	Dominance of channel-specific noise, trivial micro-clusters, or opaque state signatures without recognizable activation logic	Single-modality benchmark for myoelectric state structure
Fused side-aware family	All eleven shared domains expressed through four views each (44 domain-view blocks)	Ward hierarchical and K-means solutions screened across 2, 3, 4, and 5 states	Compactness, separation, embedding coherence, resampling stability, and interpretability	Fusion that inflates variance without improving coherent state structure, or solutions that remain unstable despite richer signal content	Primary neurorobotic representation family because it preserves both movement organization and myoelectric timing structure
Paretic-only sensitivity family	One unilateral view per shared domain (11 blocks)	Sensitivity comparison using the same low-cardinality screening logic	Concordance with the retained side-aware solution and loss of state coherence when asymmetry information is removed	Meaningful degradation in state consistency or interpretable structure after removing explicit interlimb information	Tests whether explicit asymmetry encoding adds state information beyond the paretic waveform alone
Erector-spinae-excluded sensitivity family	All shared domains except ERS-derived views	Sensitivity comparison using the same low-cardinality screening logic	State concordance, stability preservation, and retention of recognizable waveform signatures	Substantial dependence of the retained solution on one axial channel without coherent support from the remaining domains	Tests whether axial myoelectric information stabilizes or distorts the retained state structure

The public repository provides the waveform layer and methodological context for candidate state discovery, but it does not prescribe one unique cluster-number grid. The present study screened a limited low-cardinality search range chosen to balance compactness, stability, and interpretability in the modest stroke cohort. sEMG, surface electromyography; ERS, erector spinae. This table records the candidate representation families and the internal criteria used to screen them for latent gait-state discovery. The search space was intentionally limited to Ward hierarchical and K-means solutions across two to five states so that candidate partitions remained interpretable at the present sample size and compatible with waveform-level interpretation.

Interpretation of the retained solution was organized at the domain-block level rather than only at the level of reduced features. Each of the 11 shared waveform domains was treated as a distinct contribution block so that distal, proximal, and axial information could remain distinguishable after multimodal fusion. This structure is important for neurorobotics because a state space may be well separated numerically while still being too opaque to support meaningful human oversight or future adaptive-assistance logic. The prespecified domain-contribution layer is summarized in Table 4. The full computational architecture, from public repository waveforms to side-aware state discovery, interpretability, and neurorobotic relevance, is shown in Figure 1.

**Table 4:** Domain-level contribution to final gait-state separation.

Domain block	Modality	Side-aware views entering the contribution analysis	Contribution outputs expected for the retained solution	Interpretive relevance for neurorobotic state estimation
Ankle angle	Sagittal kinematics	Paretic; non-paretic; bilateral mean; side-difference	Compact waveform-level descriptive support; domain-level contribution ranking; domain-block importance mapping	Distal sagittal organization, stance-to-swing transition, and foot-clearance related state structure
Knee angle	Sagittal kinematics	Paretic; non-paretic; bilateral mean; side-difference	Compact waveform-level descriptive support; domain-level contribution ranking; domain-block importance mapping	Loading response control, mid-stance extension organization, and swing-flexion patterning
Hip angle	Sagittal kinematics	Paretic; non-paretic; bilateral mean; side-difference	Compact waveform-level descriptive support; domain-level contribution ranking; domain-block importance mapping	Proximal advancement strategy and contribution to limb progression
Pelvis angle	Sagittal kinematics	Paretic; non-paretic; bilateral mean; side-difference	Compact waveform-level descriptive support; domain-level contribution ranking; domain-block importance mapping	Global pelvic organization and proximal compensation relevant to whole-body assistance logic
Gastrocnemius normalized sEMG	Surface electromyography	Paretic; non-paretic; bilateral mean; side-difference	Compact waveform-level descriptive support; domain-level contribution ranking; domain-block importance mapping	Plantarflexor timing and push-off related activation structure
Rectus femoris normalized sEMG	Surface electromyography	Paretic; non-paretic; bilateral mean; side-difference	Compact waveform-level descriptive support; domain-level contribution ranking; domain-block importance mapping	Quadriceps-related timing around stance-to-swing transition
Vastus lateralis normalized sEMG	Surface electromyography	Paretic; non-paretic; bilateral mean; side-difference	Compact waveform-level descriptive support; domain-level contribution ranking; domain-block importance mapping	Loading-response and knee-control related activation structure
Biceps femoris normalized sEMG	Surface electromyography	Paretic; non-paretic; bilateral mean; side-difference	Compact waveform-level descriptive support; domain-level contribution ranking; domain-block importance mapping	Posterior-chain activation relevant to stance control and late-swing deceleration
Semitendinosus normalized sEMG	Surface electromyography	Paretic; non-paretic; bilateral mean; side-difference	Compact waveform-level descriptive support; domain-level contribution ranking; domain-block importance mapping	Medial hamstring timing and interlimb coordination patterns
Tibialis anterior normalized sEMG	Surface electromyography	Paretic; non-paretic; bilateral mean; side-difference	Compact waveform-level descriptive support; domain-level contribution ranking; domain-block importance mapping	Dorsiflexor activation relevant to initial contact and swing-phase limb clearance
Erector spinae normalized sEMG	Surface electromyography	Paretic; non-paretic; bilateral mean; side-difference	Compact waveform-level descriptive support; domain-level contribution ranking; domain-block importance mapping	Axial postural coordination and trunk-related contributions to whole-body gait-state organization

Quantitative contribution weights for the retained solution are reported in Supplementary material 6. The present table documents the domain blocks and interpretive targets through which those outputs are read in biomechanical and neurorobotic terms. sEMG, surface electromyography. This table identifies the signal blocks through which contribution to the retained gait-state solution is interpreted. It does not substitute for quantitative model-interpretability outputs; instead, it documents the biomechanical and myoelectric domains carried into the final interpretation layer. This distinction is important because neurorobotic usefulness depends not only on separation in latent space but also on recognizable signal logic after back-projection.

### 3.2 Low-dimensional organization of the retained fused state space

Within the strict complete-case stroke cohort (*n =* 43), the retained fused side-aware solution resolved into a three-state internal organization: State 1 (*n =* 12), State 2 (*n =* 18), and State 3 (*n =* 13). The strongest fused two-state K-means comparator had better internal compactness and resampling stability than the retained fused three-state K-means solution (silhouette 0.189 vs 0.155; bootstrap ARI 0.876 vs 0.633). The three-state solution was therefore not selected because it maximized every internal validity index. It was retained as a representation-level solution because it remained balanced, avoided trivial micro-clusters, preserved explicit side-aware kinematic-sEMG fusion, and supported more informative waveform-level interpretation than the coarser two-state alternative. In the low-dimensional embedding, State 1 occupied the left-most neighborhood, State 2 formed the broadest intermediate territory, and State 3 remained shifted to the opposite side of the retained family space. Although the confidence contours approached one another at some boundaries, they did not collapse into a single undifferentiated cloud (Figure 2).

Sensitivity analyses reinforced a restrained interpretation of this choice. Erector-spinae exclusion yielded identical subject assignments (ARI vs retained = 1.000), indicating that the retained membership was not driven by one axial channel. Robust scaling produced partial concordance (ARI = 0.785). Increasing the block cap to four components produced moderate concordance (ARI = 0.717), whereas lowering the cap to two components materially changed assignments despite acceptable internal metrics (ARI = 0.335). Collapsing the representation to a paretic-only view also changed a substantial fraction of assignments (ARI = 0.684), and kinematics-only and sEMG-only families showed limited concordance with the retained fused side-aware labels (ARI = 0.249 and 0.594, respectively). These results indicate that the retained state structure is internally reasonable but not uniquely determined; it should be read as a transparent multimodal representation choice rather than as a definitive latent phenotype.

### 3.3 Waveform-level interpretability, domain contribution, and internal robustness

Compact waveform-level descriptive summaries supported three cautious biomechanical labels for interpretation. State 1 can be described as an ankle-limited distal organization because it showed the lowest bilateral-mean ankle angle at 50% of the gait cycle and the lowest knee angle at 75%, suggesting reduced distal sagittal excursion and lower swing-flexion expression within the retained waveform space. State 2 can be described as an intermediate gastrocnemius-centered organization because it occupied the broadest latent territory and showed the highest gastrocnemius activity at 50% of the gait cycle. State 3 can be described as a dorsiflexor/swing-flexion-accentuated organization because it showed the highest tibialis anterior activity at 0, 75, and 100% and the highest knee angle at 75%. These labels are interpretive descriptors of waveform organization, not diagnostic subtypes, prognostic classes, or controller-ready states.

The domain-block contribution pattern reinforced that interpretation. Ankle angle carried the highest normalized contribution to final separation at 1.000. It was followed by vastus lateralis sEMG at 0.898, knee angle at 0.866, gastrocnemius sEMG at 0.851, and tibialis anterior sEMG at 0.840. Erector spinae sEMG, rectus femoris sEMG, hip angle, semitendinosus sEMG, biceps femoris sEMG, and pelvis angle then contributed 0.614, 0.593, 0.588, 0.473, 0.441, and 0.330, respectively. The retained separation was therefore shaped most strongly by ankle-centered sagittal mechanics together with distributed lower-limb myoelectric structure, yet it was not reducible to a single marker or a purely distal-only axis. Proximal and axial domains remained non-zero contributors, which is relevant for future adaptive assistance because state estimation may need to respond to distributed locomotor organization rather than to one isolated impairment signal (Figure 3).

To improve biomechanical readability of the retained three-state solution, Figure 4 summarizes compact waveform signatures for representative domains spanning distal kinematics, swing-related knee behavior, plantarflexor activation, and dorsiflexor activation. State 1 showed lower ankle angle at 50% of the gait cycle and lower knee angle at 75%, supporting an ankle-limited distal organization. State 2 showed the highest gastrocnemius sEMG activity at 50% of the gait cycle, supporting an intermediate gastrocnemius-centered organization. State 3 showed the highest tibialis anterior sEMG activity at 0, 75, and 100% together with the highest knee angle at 75%, supporting a dorsiflexor/swing-flexion-accentuated organization. These waveform signatures are presented as descriptive support for representation-level interpretation only.

The retained solution was not accepted on low-dimensional appearance alone. The full mapping between repository variables, domain labels, and representation views is itemized in Supplementary material 1, and the conservative preprocessing, scaling, feature-engineering pathway, sEMG-normalization convention, and side-difference sign are expanded in Supplementary material 2. The broader grid of candidate families, clustering methods, state numbers, two-state comparator, and constrained side-averaged able-bodied comparator is documented in Supplementary material 3. Stability, resampling, component-retention sensitivity, paretic-only sensitivity, ERS-exclusion sensitivity, and modality-specific baselines are reported in Supplementary material 4.

The interpretability and reproducibility layers were likewise made explicit. Compact descriptive summaries and state-descriptor labels supporting waveform-level interpretation are reported in Supplementary material 5, domain-level contribution outputs for the retained solution are reported in Supplementary material 6, and the subject-level state-assignment matrix, excluded complete-case participants, and included-versus-excluded available-kinematic comparison are reported in Supplementary material 7. A deterministic execution path based on public repository exports and standard open-source software is described in Supplementary material 8, together with the exact workflow specifications recoverable from the archived manuscript materials and the stochastic implementation metadata that were not exhaustively enumerated at manuscript level. Accordingly, the reproducibility claim of the present study is limited but concrete. The representation workflow can be reconstructed from the public waveform layer, whereas exact stochastic outputs require executable-code metadata.

## 4 Discussion

The main contribution of this study is not the proposal of a new clinical label, nor the validation of a wearable controller, but the demonstration that a side-aware multimodal waveform space derived from public post-stroke gait exports can support an internally coherent and interpretable state representation. That contribution sits within a broader movement in gait science toward richer computational representations that preserve temporal structure instead of collapsing locomotion into a handful of scalar summary scores. Recent reviews of machine-learning methods in marker-based clinical gait analysis have highlighted both the opportunity and the risk of this shift: richer models can reveal structure missed by conventional analyses, but they can become detached from biomechanical interpretation if compression and classification are treated as ends in themselves (Dibbern et al., 2025). A parallel review of non-linear biomechanical assessment in chronic stroke reached a similar conclusion, emphasizing that complex models are most useful when they remain linked to recognizable locomotor organization rather than to mathematically elegant but clinically opaque latent variables (Freitas et al., 2024). The present results address that challenge by retaining waveform anchoring, explicit side awareness, and domain-block interpretation.

The retained three-state solution should be interpreted through the trade-off that produced it. The strongest fused two-state comparator was numerically stronger for compactness and resampling stability, and this is now reported explicitly. The reason for retaining the three-state solution was not superiority on every internal index, but representation utility: the three-state structure preserved a balanced multimodal side-aware organization, avoided trivial micro-clusters, and separated waveform signatures that could be described in biomechanical terms. The two-state solution is therefore best viewed as a stronger but coarser comparator, whereas the three-state solution is an internally retained representation that better preserves within-cohort heterogeneity. Neural-network analysis of gait-cycle kinematics and personalized causal-network modelling have likewise suggested that post-stroke gait organization can differ across individuals rather than collapsing into one abnormal prototype (Kuch et al., 2025; Nishi et al., 2024). Nevertheless, the present states are not externally validated phenotypes and should not be read as diagnostic subtypes.

The preference for the fused family over kinematics-only or sEMG-only families is also conceptually important. Post-stroke gait can show similar joint trajectories with different underlying muscle-activation strategies, just as comparable sEMG bursts can accompany different kinematic compensations. Recent comparative work has shown that subphase-specific myoelectric patterns in chronic stroke can reveal gait disorder structure that is not captured by gross temporal descriptors alone (Hohsoh et al., 2024). At the same time, consensus-oriented work on surface electromyography within clinical gait analysis has reiterated that sEMG is most informative when interpreted in relation to movement context rather than as an isolated channel set (Reisig et al., 2025). In the present study, modality-specific baselines did not reproduce the retained fused side-aware labels with high concordance, and the paretic-only sensitivity family reassigned a substantial fraction of subjects. These findings support the representational value of combining side-aware kinematics with normalized sEMG, while also showing that the retained solution is method-dependent and must be validated externally.

The explicit inclusion of paretic, non-paretic, bilateral-mean, and paretic-minus-non-paretic side-difference views appears especially valuable in the context of post-stroke locomotion. Recent biomechanical work in subacute ischemic stroke has reinforced that gait abnormalities are distributed across distal, proximal, and trunk-related mechanics rather than confined to one joint (Skvortsov et al., 2025). Work on temporal gait asymmetry has further shown that observed asymmetry reflects both impairment and compensation, such that the non-paretic limb can carry explanatory information rather than serving as a passive comparator (Mizuta et al., 2025). Narrative synthesis of stiff-knee mechanisms likewise underscores that post-stroke gait patterns often arise from interacting deficits and compensations rather than from one isolated pathological driver (Krajewski et al., 2025). The present side-aware framework operationalizes that insight by representing unilateral behavior, contralateral organization, global locomotor patterning, and directional interlimb difference in the same feature space.

These considerations are directly relevant to lower-limb neurorobotics at the representation-design stage. Recent reviews of artificial-intelligence-based methods, sensor technology, control strategies, and clinical effectiveness in rehabilitation exoskeletons consistently point to the same unmet need: assistance policies must be conditioned on states that are informative enough to adapt yet transparent enough to supervise (Coser et al., 2024; Yao et al., 2024; de Miguel-Fernandez et al., 2023; Baud et al., 2021). The present study does not provide such a policy. It asks what kind of internal state space may be worth estimating before a policy is designed. A cautious future mapping can be stated as follows. A State 1-like organization might motivate closer monitoring of distal sagittal mechanics, ankle-foot clearance, or distal assistance windows. A State 2-like organization might motivate attention to gastrocnemius timing and push-off-related assistance windows. A State 3-like organization might motivate monitoring of dorsiflexor activity, swing-phase support, and avoidance of excessive knee or ankle constraint. This mapping is conceptual and requires prospective validation; it should not be used as a controller rule.

The domain-contribution pattern also suggests a possible deployable sensing pathway, but only as a hypothesis for future work. A reduced sensor set could prioritize ankle-angle estimation, knee-angle estimation, and sEMG from vastus lateralis, gastrocnemius, and tibialis anterior, with optional rectus femoris, erector spinae, or pelvis/trunk inertial sensing when axial or proximal compensation is clinically relevant. Such a pathway could be implemented with joint encoders or wearable inertial units for ankle and knee kinematics and surface electrodes for the three dominant lower-limb myoelectric channels. However, the present dataset does not test wearable placement, streaming latency, online filtering, embedded computation, electrode stability, or controller switching. Any expectation of low-latency state estimation therefore remains a design target rather than a result of this study. Reviews of portable exoskeletons, wearable gait robotics, and locomotor exoskeleton translation emphasize exactly this gap between interpretable sensing concepts and deployment-ready systems (Jiang et al., 2024; Cha et al., 2025; Siviy et al., 2023; Lee et al., 2024).

The clinical value of the retained states should also be framed carefully. The states should not be read as diagnostic subtypes, clinical biomarkers, prognostic groups, or severity categories. Their value is instead methodological and preparatory: they provide a transparent way to organize side-dependent waveform information before future clinical or robotic validation. Human-in-the-loop optimization studies increasingly use electrophysiological or performance-based objectives to tune wearable assistance, and emerging work has shown that EMG-informed objective functions, sEMG-biofeedback trajectories, deep-learning phase identification, and multi-gait optimization strategies can improve adaptation to user-specific locomotor conditions (Diaz et al., 2024; Li et al., 2024; Soldi et al., 2025; Jing et al., 2024). Those advances presuppose that the underlying state variables are meaningful. A poorly defined state can be optimized efficiently and still remain behaviorally shallow. The present results argue for a complementary agenda in which personalization begins with interpretable representation design.

Several limitations define the appropriate scope of the findings. The final fused state solution was derived from a small strict complete-case stroke cohort (*n =* 43), with seven stroke participants excluded from fused state discovery because paired lower-limb normalized sEMG was incomplete. The study depended on public preprocessed spreadsheet waveforms and did not revisit raw C3D processing, stride-level variability, force-plate synchronization, electrode-level signal quality, or alternative EMG-normalization choices. No imputation of missing sEMG was performed because that would have synthesized waveform channels absent from the public analytic layer. The able-bodied workbook was useful for shared-domain eligibility and descriptive reference context, but it was not structurally equivalent to the stroke workbook for side-aware left–right modelling. The retained three-state solution was internally supported but not uniquely determined, and the component-retention sensitivity analysis showed substantial reassignment when the block cap was reduced to two components. There was no external validation, no prospective clinical outcome linkage, no controller implementation, no hardware experiment, no online state estimator, and no latency test. Future work should therefore test whether side-aware multimodal states can be estimated from deployment-feasible sensors, reproduced in independent cohorts, linked to clinical outcomes, and incorporated into adaptive control without sacrificing interpretability or safety (Ye et al., 2026; Gao et al., 2025; Nicora et al., 2025; Schopp et al., 2026).

## 5 Conclusion

In this public-dataset secondary analysis, a side-aware fused kinematic-sEMG representation of post-stroke gait supported an interpretable three-state internal organization within the strict complete-case stroke cohort, together with compact waveform-level interpretation and distributed domain contributions led by ankle angle, lower-limb sEMG, and knee-angle structure. The retained three-state solution was selected as a representation-level compromise rather than as the numerically strongest partition, because the strongest fused two-state comparator was more compact and more stable but coarser.

This contribution remains exploratory and representational. The study does not validate a device, a decoder, a real-time state estimator, a clinical biomarker, or a control strategy, and the retained states should not be treated as externally generalizable phenotypes. Its translational value is preparatory: it shows that a public waveform layer can support transparent latent-state discovery, domain-level interpretation, and sensitivity-oriented screening. The next step is prospective validation in independent datasets and online testing to determine whether similarly side-aware multimodal states can be estimated from deployable sensors and used to improve human-robot adaptation without sacrificing interpretability or safety.

## Data Availability

The datasets presented in this study can be found in online repositories. The names of the repository/repositories and accession number(s) can be found at: https://doi.org/10.6084/m9.figshare.c.6503791.v1.
