# Supplementary material for: Interpretable side-aware kinematic-sEMG gait-state representations relevant to adaptive neurorobotic assistance after stroke: a public-dataset study
Source: Front Neurorobot. 2026 May 25;20:1863916. doi: 10.3389/fnbot.2026.1863916 (PMC13243435; doi:10.3389/fnbot.2026.1863916)
Supplement: Supplementary file 1 [file Data_Sheet_1.docx]

**Supplementary Material 1.** Full domain-view dictionary and analytic variable mapping raw-export completeness is reported as a repository property and should not be equated automatically with downstream retained-state membership. This supplementary material defines the shared waveform space used in the neurorobotics manuscript and maps repository variable names to manuscript terminology. The first table records the domain dictionary and availability profile, whereas the second table defines the representation views derived from the stroke-side exports. Together, these tables provide the vocabulary needed to trace every later representation, stability, and explainability step back to the public repository layer.

**A. Shared-domain dictionary**

| **Repository label** | **Manuscript domain label** | **Signal family** | **Unit or scale** | **Able-bodied workbook label** | **Stroke workbook label** | **Raw-export completeness** |
| --- | --- | --- | --- | --- | --- | --- |
| AnkleAngles | Ankle angle | Sagittal kinematics | degrees | AnkleAngles | Pside_AnkleAngles / Nside_AnkleAngles | AB 138/138; P 50/50; N 50/50; paired 50/50 |
| KneeAngles | Knee angle | Sagittal kinematics | degrees | KneeAngles | Pside_KneeAngles / Nside_KneeAngles | AB 138/138; P 50/50; N 50/50; paired 50/50 |
| HipAngles | Hip angle | Sagittal kinematics | degrees | HipAngles | Pside_HipAngles / Nside_HipAngles | AB 138/138; P 50/50; N 50/50; paired 50/50 |
| PelvisAngles | Pelvis angle | Sagittal kinematics | degrees | PelvisAngles | Pside_PelvisAngles / Nside_PelvisAngles | AB 138/138; P 50/50; N 50/50; paired 50/50 |
| GASnorm | Gastrocnemius normalized sEMG | Surface electromyography | Normalized repository amplitude | GASnorm | Pside_GASnorm / Nside_GASnorm | AB 109/138; P 43/50; N 44/50; paired 43/50 |
| RFnorm | Rectus femoris normalized sEMG | Surface electromyography | Normalized repository amplitude | RFnorm | Pside_RFnorm / Nside_RFnorm | AB 108/138; P 43/50; N 44/50; paired 43/50 |
| VLnorm | Vastus lateralis normalized sEMG | Surface electromyography | Normalized repository amplitude | VLnorm | Pside_VLnorm / Nside_VLnorm | AB 109/138; P 43/50; N 44/50; paired 43/50 |
| BFnorm | Biceps femoris normalized sEMG | Surface electromyography | Normalized repository amplitude | BFnorm | Pside_BFnorm / Nside_BFnorm | AB 106/138; P 43/50; N 44/50; paired 43/50 |
| STnorm | Semitendinosus normalized sEMG | Surface electromyography | Normalized repository amplitude | STnorm | Pside_STnorm / Nside_STnorm | AB 108/138; P 43/50; N 44/50; paired 43/50 |
| TAnorm | Tibialis anterior normalized sEMG | Surface electromyography | Normalized repository amplitude | TAnorm | Pside_TAnorm / Nside_TAnorm | AB 107/138; P 43/50; N 44/50; paired 43/50 |
| ERSnorm | Erector spinae normalized sEMG | Surface electromyography | Normalized repository amplitude | ERSnorm | Pside_ERSnorm / Nside_ERSnorm | AB 106/138; P 46/50; N 46/50; paired 46/50 |

Note. The completeness counts reported here describe native spreadsheet-export availability. They do not by themselves determine final latent-state membership, which depends on the executed representation workflow. Because the able-bodied spreadsheet layer is side-averaged, no direct left-right difference export is available for the reference cohort in this public analytic layer.

**B. Representation-view dictionary**

| **Representation view** | **How the view is obtained** | **Able-bodied reference-layer availability** | **Stroke-layer availability** | **Role in side-aware state representation** |
| --- | --- | --- | --- | --- |
| Paretic | Native Pside export for each shared stroke domain | No direct side-homologous view in the able-bodied spreadsheet layer | Yes | Preserves unilateral abnormality on the clinically affected side |
| Non-paretic | Native Nside export for each shared stroke domain | No direct side-homologous view in the able-bodied spreadsheet layer | Yes | Preserves contralateral organization and compensation |
| Bilateral mean | Arithmetic mean of the paired paretic and non-paretic waveforms | The able-bodied workbook provides one side-averaged waveform per domain | Yes, when both stroke sides are complete at the raw-export level | Captures global locomotor organization while reducing explicit side dependence |
| Side-difference | Paretic minus non-paretic waveform | No homologous left-right difference view is directly available in the able-bodied spreadsheet layer | Yes, when both stroke sides are complete at the raw-export level | Makes asymmetry an explicit representation target for state discovery |

**Note.** The side-difference view is a derived asymmetry construct rather than a native repository field. Because the able-bodied spreadsheet layer is side-averaged, no direct left-right difference export is available for the reference cohort in this public analytic layer. **Throughout the manuscript and supplementary materials, the side-difference waveform is defined directionally as paretic minus non-paretic. This convention preserves the sign of interlimb asymmetry and avoids converting asymmetry into an unsigned magnitude.**

**Acronyms.** sEMG = surface electromyography; AB = able-bodied; GAS = gastrocnemius; RF = rectus femoris; VL = vastus lateralis; BF = biceps femoris; ST = semitendinosus; TA = tibialis anterior; ERS = erector spinae.
