# Supplementary material for: Interpretable side-aware kinematic-sEMG gait-state representations relevant to adaptive neurorobotic assistance after stroke: a public-dataset study
Source: Front Neurorobot. 2026 May 25;20:1863916. doi: 10.3389/fnbot.2026.1863916 (PMC13243435; doi:10.3389/fnbot.2026.1863916)
Supplement: Supplementary file 2 [file Data_Sheet_2.docx]

**Supplementary Material 2.** Detailed preprocessing, scaling, and feature-engineering protocol. This supplementary material expands the methodological steps that connect the public spreadsheet exports to the final representation families used for latent gait-state discovery. The first table records the main processing sequence, the second clarifies which operations were intentionally not performed, and the third summarizes the descriptor families used to support explainability. The emphasis is on reproducible, conservative handling of the repository waveforms rather than on aggressive signal manipulation.

**A. Stepwise preprocessing and representation protocol**

| **Processing step** | **Operational rule** | **Output object** | **Key restriction or rationale** |
| --- | --- | --- | --- |
| Repository import | All spreadsheet columns are imported exactly as distributed in the public repository | Participant-level domain waveforms | No hidden preprocessing is inserted before domain restriction |
| Shared-space restriction | Analysis is restricted to the eleven domains that are genuinely shared across the able-bodied and stroke spreadsheet exports | Comparable cross-cohort signal space | Kinetics and ground-reaction-force variables are excluded because they are not available in the public stroke spreadsheet export |
| Numeric integrity screen | Each waveform is checked for full 1001-point length, missing samples, and constant-value artifacts | Analysis-ready domain flags | A domain-view is treated as complete only when the exported curve is non-constant and numerically complete |
| Side-aware view construction | Each shared stroke domain is expanded into paretic, non-paretic, bilateral mean, and side-difference views when paired side data are present | Four complementary views per domain | Bilateral mean and side-difference require paired stroke-side availability |
| Within-cohort pointwise standardization | Each domain-view waveform is centered and scaled at the pointwise level within cohort before functional reduction | Standardized domain-view inputs | Robust scaling is reserved for sensitivity analysis |
| Functional reduction | Dimensionality is reduced within each domain-view combination rather than across all curves at once | Compact coefficient sets | This preserves domain identity while controlling overparameterization |
| Explainability support descriptors | Lower-complexity summaries are generated alongside the functional representation | Auditable scalar support features | These descriptors support interpretation and surrogate explainability rather than replacing the primary representation |
| Representation-family assembly | Reduced features are grouped into kinematics-only, sEMG-only, and fused state spaces | Candidate latent-state input families | This assembly is the basis for comparing multimodal fusion against single-modality representations |
| **Repository sEMG normalization** | **Normalized sEMG amplitudes retained exactly as exported; no additional normalization applied** | **Repository-normalized sEMG waveforms** | **Preserves the public analytic layer and avoids introducing non-public signal-processing assumptions** |

**B. Operations intentionally not performed**

| **Operation** | **Status** | **Reason for omission in the public spreadsheet workflow** |
| --- | --- | --- |
| Low-pass refiltering | Not performed | The public waveform export was treated as the final functional object |
| Gait-cycle realignment | Not performed | Re-segmentation without the full source structures would materially alter waveform morphology |
| Derivative expansion | Not performed | Higher-order transforms were intentionally avoided in the spreadsheet-based public workflow |
| Raw force-plate reinterpretation | Not performed | Force and moment variables were not available in the public stroke spreadsheet layer used for neurorobotic state modelling |
| Raw EMG re-normalization | Not performed | The study retained the normalization and export logic already encoded in the public repository |

**C. Explainability-support descriptor families**

| **Descriptor family** | **Operational role** | **Interpretive purpose** |
| --- | --- | --- |
| Waveform level | Mean or level-centered summaries extracted from the original curve | Supports interpretation of sustained offset-like behavior |
| Peak behavior | Peak, trough, or extremum-oriented summaries | Supports interpretation of amplitude-dominant deviations |
| Integrated magnitude | Area-like summaries over the gait cycle | Supports interpretation of cumulative activation or excursion burden |
| Timing-related summaries | Phase-sensitive or centroid-like timing descriptors | Supports interpretation of temporal redistribution within the cycle |
| Side-difference burden | Asymmetry-oriented summaries derived from paired views | Supports interpretation of interlimb imbalance relevant to adaptive assistance logic |

Note. The primary analysis used within-cohort pointwise standardization applied within each domain-view combination before dimensionality reduction. Robust scaling was examined as a sensitivity analysis. The able-bodied cohort informed the shared-domain restriction and descriptive context, but it was not used as the primary scaling anchor for the retained numerical state-discovery layer. **The public stroke workbook specifies that normalized EMG was normalized to the maximum of each individual muscle during the gait cycle. The present analysis retained these repository-normalized amplitudes exactly as exported and did not perform raw EMG re-normalization, MVC scaling, reference-contraction normalization, or additional envelope processing.**

**Acronyms.** sEMG = surface electromyography; FPCA = functional principal component analysis; ERS = erector spinae.
