# Supplementary material for: Interpretable side-aware kinematic-sEMG gait-state representations relevant to adaptive neurorobotic assistance after stroke: a public-dataset study
Source: Front Neurorobot. 2026 May 25;20:1863916. doi: 10.3389/fnbot.2026.1863916 (PMC13243435; doi:10.3389/fnbot.2026.1863916)
Supplement: Supplementary file 3 [file Data_Sheet_3.docx]

**Supplementary Material 3. Candidate state solutions, tuning grid, and internal validity indices**

This supplementary file reports the numerical screening outputs obtained directly from the original public waveform exports. The analysis used the strict fused complete-case stroke cohort (n = 43), the 11 shared waveform domains, four side-aware views per domain, within-cohort pointwise standardization, block-wise principal-component compression to 90% cumulative variance with a cap of three components per block, and family-level compression to 90% cumulative variance with a cap of eight components. For transparency, it also includes a direct comparison between the strongest fused two-state comparator and the retained fused three-state solution.

**Table S3.1. Candidate solution screening across representation families, clustering methods, and state numbers.**

| **Family** | **Method** | **States** | **Cluster sizes (sorted)** | **Silhouette** | **Calinski-Harabasz** | **Davies-Bouldin** | **Bootstrap ARI mean** | **Bootstrap ARI SD** | **Minimum cluster size** |
| --- | --- | --- | --- | --- | --- | --- | --- | --- | --- |
| Kinematics-only | Ward hierarchical | 2 | 21,22 | 0.161 | 7.98 | 2.148 | 0.488 | 0.29 | 21 |
| Kinematics-only | Ward hierarchical | 3 | 10,11,22 | 0.184 | 8.41 | 1.732 | 0.605 | 0.252 | 10 |
| Kinematics-only | Ward hierarchical | 4 | 5,10,11,17 | 0.133 | 7.57 | 1.623 | 0.525 | 0.172 | 5 |
| Kinematics-only | Ward hierarchical | 5 | 1,5,10,10,17 | 0.141 | 7.27 | 1.322 | 0.566 | 0.189 | 1 |
| Kinematics-only | K-means | 2 | 21,22 | 0.182 | 9.93 | 1.926 | 0.67 | 0.24 | 21 |
| Kinematics-only | K-means | 3 | 10,14,19 | 0.18 | 9.12 | 1.707 | 0.633 | 0.195 | 10 |
| Kinematics-only | K-means | 4 | 6,7,13,17 | 0.166 | 8.18 | 1.549 | 0.535 | 0.193 | 6 |
| Kinematics-only | K-means | 5 | 1,8,8,10,16 | 0.164 | 7.7 | 1.357 | 0.554 | 0.184 | 1 |
| sEMG-only | Ward hierarchical | 2 | 12,31 | 0.164 | 9.46 | 1.699 | 0.609 | 0.291 | 12 |
| sEMG-only | Ward hierarchical | 3 | 4,12,27 | 0.176 | 7.7 | 1.66 | 0.447 | 0.197 | 4 |
| sEMG-only | Ward hierarchical | 4 | 4,6,12,21 | 0.1 | 7.07 | 1.609 | 0.479 | 0.155 | 4 |
| sEMG-only | Ward hierarchical | 5 | 4,6,6,12,15 | 0.104 | 6.73 | 1.64 | 0.49 | 0.171 | 4 |
| sEMG-only | K-means | 2 | 18,25 | 0.173 | 10.21 | 1.898 | 0.825 | 0.152 | 18 |
| sEMG-only | K-means | 3 | 13,13,17 | 0.152 | 8.51 | 1.951 | 0.542 | 0.23 | 13 |
| sEMG-only | K-means | 4 | 3,12,12,16 | 0.158 | 7.82 | 1.631 | 0.521 | 0.167 | 3 |
| sEMG-only | K-means | 5 | 4,8,10,10,11 | 0.14 | 7.33 | 1.649 | 0.501 | 0.155 | 4 |
| Fused kinematic-sEMG | Ward hierarchical | 2 | 12,31 | 0.172 | 10.19 | 1.611 | 0.727 | 0.304 | 12 |
| Fused kinematic-sEMG | Ward hierarchical | 3 | 12,14,17 | 0.144 | 8.8 | 1.975 | 0.616 | 0.189 | 12 |
| Fused kinematic-sEMG | Ward hierarchical | 4 | 4,10,12,17 | 0.16 | 7.93 | 1.691 | 0.634 | 0.173 | 4 |
| Fused kinematic-sEMG | Ward hierarchical | 5 | 2,4,8,12,17 | 0.179 | 7.5 | 1.439 | 0.655 | 0.151 | 2 |
| Fused kinematic-sEMG | K-means | 2 | 20,23 | 0.189 | 11.73 | 1.784 | 0.876 | 0.133 | 20 |
| Fused kinematic-sEMG | K-means | 3 | 12,13,18 | 0.155 | 9.48 | 1.916 | 0.633 | 0.221 | 12 |
| Fused kinematic-sEMG | K-means | 4 | 3,12,13,15 | 0.167 | 8.38 | 1.656 | 0.609 | 0.162 | 3 |
| Fused kinematic-sEMG | K-means | 5 | 6,7,8,10,12 | 0.154 | 7.63 | 1.637 | 0.548 | 0.162 | 6 |

**Table S3.2. Comparison of all three-state candidates and rationale for retention.**

| **Family** | **Method** | **Cluster sizes (sorted)** | **Silhouette** | **Davies-Bouldin** | **Bootstrap ARI mean** | **Retention note** |
| --- | --- | --- | --- | --- | --- | --- |
| Kinematics-only | Ward hierarchical | 10,11,22 | 0.184 | 1.732 | 0.605 |  |
| Kinematics-only | K-means | 10,14,19 | 0.18 | 1.707 | 0.633 | Close 3-state alternative with slightly higher compactness. |
| sEMG-only | Ward hierarchical | 4,12,27 | 0.176 | 1.66 | 0.447 |  |
| sEMG-only | K-means | 13,13,17 | 0.152 | 1.951 | 0.542 | Less stable than fused and kinematics-only 3-state alternatives. |
| Fused kinematic-sEMG | Ward hierarchical | 12,14,17 | 0.144 | 1.975 | 0.616 | Balanced 3-state fused alternative with similar internal stability. |
| Fused kinematic-sEMG | K-means | 12,13,18 | 0.155 | 1.916 | 0.633 | Retained as the primary multimodal 3-state solution. |

Note. In this analysis, the highest compactness and stability values occurred in several two-state solutions. The primary retained solution was therefore not chosen because it maximized every compactness index. It was retained because it remained a balanced three-state multimodal solution without micro-clusters, preserved explicit side-aware kinematic-sEMG fusion, and remained sufficiently stable to support waveform-level interpretation. Table S3.3 isolates the direct comparison between the strongest fused two-state candidate and the retained fused three-state solution.

**Table S3.3. Direct comparison of the strongest fused two-state comparator and the retained fused three-state solution.**

| Solution | Cluster sizes | Silhouette | Bootstrap ARI mean | Direct interpretation |
| --- | --- | --- | --- | --- |
| Strongest fused comparator (fused kinematic-sEMG, K-means, 2 states) | 20,23 | 0.189 | 0.876 | Numerically strongest fused solution, but coarser than the retained representation goal. |
| Retained solution (fused kinematic-sEMG, K-means, 3 states) | 12,18,13 | 0.155 | 0.633 | Lowest-cardinality multimodal solution preserving side-aware fusion and waveform-level interpretability. |

Note. The direct comparison isolates the central methodological trade-off documented in the manuscript. Sensitivity results reported in Supplementary Material 4 apply to the retained solution; direct two-state-versus-retained label concordance was not part of the archived screening tables.

**Additional comparator. Because the public able-bodied workbook provides one side-averaged waveform per domain and does not provide homologous left-right exports, a direct side-aware healthy-control test was not possible. A constrained side-averaged able-bodied comparator was therefore used only as a descriptive check. Forced Ward partitions in the 102 complete able-bodied cases did not reproduce a stable side-aware stroke-like structure; the two-state comparator had low bootstrap stability despite acceptable silhouette, and the three-state comparator showed lower silhouette and only modest bootstrap stability.**

**Table S3.4. Constrained side-averaged able-bodied comparator using the 102 complete able-bodied cases.**

| **Family** | **Method** | **States** | **Cluster sizes (sorted)** | **Silhouette** | **Calinski-Harabasz** | **Davies-Bouldin** | **Bootstrap ARI mean** | **Minimum cluster size** |
| --- | --- | --- | --- | --- | --- | --- | --- | --- |
| **Able-bodied side-averaged** | **Ward hierarchical** | **2** | **20,82** | **0.226** | **20.01** | **1.792** | **0.084** | **20** |
| **Able-bodied side-averaged** | **Ward hierarchical** | **3** | **20,28,54** | **0.115** | **18.43** | **1.998** | **0.340** | **20** |
| **Able-bodied side-averaged** | **Ward hierarchical** | **4** | **7,13,28,54** | **0.138** | **17.68** | **1.706** | **0.353** | **7** |
| **Able-bodied side-averaged** | **Ward hierarchical** | **5** | **7,11,13,28,43** | **0.133** | **16.40** | **1.721** | **0.371** | **7** |

**Note. This comparator is not a direct validation of stroke specificity because the able-bodied spreadsheet export is side-averaged and cannot reproduce paretic, non-paretic, bilateral-mean, and paretic-minus-non-paretic views. It is included to use the able-bodied cohort more transparently while avoiding a false side-aware comparison.**
