# Supplementary material for: Interpretable side-aware kinematic-sEMG gait-state representations relevant to adaptive neurorobotic assistance after stroke: a public-dataset study
Source: Front Neurorobot. 2026 May 25;20:1863916. doi: 10.3389/fnbot.2026.1863916 (PMC13243435; doi:10.3389/fnbot.2026.1863916)
Supplement: Supplementary file 4 [file Data_Sheet_4.docx]

**Supplementary Material 4. Stability, resampling, and sensitivity results across representation families**

This supplementary file reports the stability and sensitivity outputs for the retained solution. The retained solution was the fused side-aware three-state K-means solution fitted in the strict complete-case stroke cohort (n = 43). Bootstrap stability is reported as the mean adjusted Rand index (ARI) under repeated 80% subsampling. Sensitivity concordance is reported as the ARI between each challenged solution and the retained labels on the same subject set.

**Table S4.1. Internal screening across fused-family candidate solutions.**

| **Method** | **States** | **Cluster sizes (sorted)** | **Silhouette** | **Calinski-Harabasz** | **Davies-Bouldin** | **Bootstrap ARI mean** | **Bootstrap ARI SD** | **Minimum cluster size** |
| --- | --- | --- | --- | --- | --- | --- | --- | --- |
| Ward hierarchical | 2 | 12,31 | 0.172 | 10.19 | 1.611 | 0.727 | 0.304 | 12 |
| Ward hierarchical | 3 | 12,14,17 | 0.144 | 8.8 | 1.975 | 0.616 | 0.189 | 12 |
| Ward hierarchical | 4 | 4,10,12,17 | 0.16 | 7.93 | 1.691 | 0.634 | 0.173 | 4 |
| Ward hierarchical | 5 | 2,4,8,12,17 | 0.179 | 7.5 | 1.439 | 0.655 | 0.151 | 2 |
| K-means | 2 | 20,23 | 0.189 | 11.73 | 1.784 | 0.876 | 0.133 | 20 |
| K-means | 3 | 12,13,18 | 0.155 | 9.48 | 1.916 | 0.633 | 0.221 | 12 |
| K-means | 4 | 3,12,13,15 | 0.167 | 8.38 | 1.656 | 0.609 | 0.162 | 3 |
| K-means | 5 | 6,7,8,10,12 | 0.154 | 7.63 | 1.637 | 0.548 | 0.162 | 6 |

**Table S4.2. Sensitivity analyses relative to the retained fused side-aware three-state solution.**

| **Scenario** | **Cluster sizes** | **Silhouette** | **Bootstrap ARI mean** | **Bootstrap ARI SD** | **ARI vs retained** | **Family components** | **Family cumulative variance** | **Pre-family features** |
| --- | --- | --- | --- | --- | --- | --- | --- | --- |
| Retained fused side-aware | 12,18,13 | 0.155 | 0.633 | 0.221 | 1.0 | 8 | 0.603 | 123 |
| Robust scaling | 12,19,12 | 0.14 | 0.524 | 0.235 | 0.785 | 8 | 0.596 | 123 |
| Lower block cap (2 PCs) | 17,10,16 | 0.162 | 0.764 | 0.186 | 0.335 | 8 | 0.651 | 85 |
| Higher block cap (4 PCs) | 12,20,11 | 0.154 | 0.557 | 0.285 | 0.717 | 8 | 0.559 | 154 |
| ERS excluded | 12,18,13 | 0.152 | 0.624 | 0.215 | 1.0 | 8 | 0.606 | 111 |
| Paretic-only view | 13,15,15 | 0.176 | 0.856 | 0.134 | 0.684 | 8 | 0.735 | 31 |
| Kinematics-only family | 14,10,19 | 0.18 | 0.633 | 0.195 | 0.249 | 8 | 0.861 | 39 |
| sEMG-only family | 17,13,13 | 0.152 | 0.542 | 0.23 | 0.594 | 8 | 0.63 | 84 |

*Interpretive note. ERS exclusion yielded identical subject assignments to the retained solution (ARI vs retained = 1.000), indicating that the lower-limb and side-aware signal structure drove the retained state membership. Lowering the block cap to two components increased bootstrap stability but materially changed subject assignments (ARI vs retained = 0.335), whereas paretic-only representations increased compactness and stability while still reassigning a substantial fraction of subjects (ARI vs retained = 0.684). These results support interpreting the retained solution as internally reasonable but not uniquely determined.*
