# Supplementary material for: Interpretable side-aware kinematic-sEMG gait-state representations relevant to adaptive neurorobotic assistance after stroke: a public-dataset study
Source: Front Neurorobot. 2026 May 25;20:1863916. doi: 10.3389/fnbot.2026.1863916 (PMC13243435; doi:10.3389/fnbot.2026.1863916)
Supplement: Supplementary file 5 [file Data_Sheet_5.docx]

**Supplementary Material 5. Compact descriptive support for waveform-level interpretation of the retained gait states**

This supplementary material documents the domain set, compact waveform summaries, and reporting conventions used to support waveform-level interpretation of the retained states. The first table lists the shared domains considered for extended reporting. The second table reports state-wise bilateral-mean descriptive summaries at five gait-cycle anchors for a compact set of representative domains.

**A. Domain set used for waveform-level interpretability support**

| **Domain** | **Views used for extended reporting** | **Native unit** | **Why the domain matters for state interpretation** |
| --- | --- | --- | --- |
| Ankle angle | Paretic and non-paretic views, with bilateral mean and side-difference when informative | degrees | Distal sagittal organization and foot-clearance strategy |
| Knee angle | Paretic and non-paretic views, with bilateral mean and side-difference when informative | degrees | Stance control and swing-flexion patterning |
| Hip angle | Paretic and non-paretic views, with bilateral mean and side-difference when informative | degrees | Proximal advancement strategy |
| Pelvis angle | Paretic and non-paretic views, with bilateral mean and side-difference when informative | degrees | Global pelvic organization and compensation |
| Gastrocnemius normalized sEMG | Paretic and non-paretic views, with bilateral mean and side-difference when informative | normalized amplitude | Push-off related activation timing |
| Rectus femoris normalized sEMG | Paretic and non-paretic views, with bilateral mean and side-difference when informative | normalized amplitude | Quadriceps-related timing around stance-to-swing transition |
| Vastus lateralis normalized sEMG | Paretic and non-paretic views, with bilateral mean and side-difference when informative | normalized amplitude | Loading-response activation structure |
| Biceps femoris normalized sEMG | Paretic and non-paretic views, with bilateral mean and side-difference when informative | normalized amplitude | Posterior-chain contribution to state structure |
| Semitendinosus normalized sEMG | Paretic and non-paretic views, with bilateral mean and side-difference when informative | normalized amplitude | Medial hamstring coordination structure |
| Tibialis anterior normalized sEMG | Paretic and non-paretic views, with bilateral mean and side-difference when informative | normalized amplitude | Initial-contact and swing-phase dorsiflexor behavior |
| Erector spinae normalized sEMG | Paretic and non-paretic views, with bilateral mean and side-difference when informative | normalized amplitude | Axial postural coordination structure |

**B. Compact bilateral-mean descriptive summaries for representative waveform domains**

Table S5.2 reports mean (SD) values across subjects within each retained state at 0%, 25%, 50%, 75%, and 100% of the gait cycle for four representative domains used to summarize waveform-level differences across the retained states. These summaries provide compact numerical support in native domain units.

| **Domain** | **Gait cycle (%)** | **State 1 mean (SD)** | **State 2 mean (SD)** | **State 3 mean (SD)** |
| --- | --- | --- | --- | --- |
| Ankle angle | 0 | 2.292 (4.858) | 0.967 (4.292) | -0.660 (4.199) |
| Ankle angle | 25 | 7.493 (4.887) | 7.547 (5.667) | 7.281 (3.273) |
| Ankle angle | 50 | 10.987 (4.348) | 14.474 (5.138) | 13.553 (2.709) |
| Ankle angle | 75 | 8.429 (2.329) | 4.191 (5.501) | 2.182 (3.933) |
| Ankle angle | 100 | 2.252 (5.016) | 0.799 (4.544) | -1.307 (4.156) |
| Knee angle | 0 | 22.942 (7.733) | 17.937 (7.008) | 15.695 (5.564) |
| Knee angle | 25 | 15.599 (11.415) | 16.573 (9.866) | 16.812 (6.892) |
| Knee angle | 50 | 16.393 (11.610) | 14.251 (8.761) | 12.993 (7.215) |
| Knee angle | 75 | 28.837 (10.936) | 44.778 (11.028) | 54.747 (10.268) |
| Knee angle | 100 | 23.004 (7.791) | 18.008 (7.097) | 15.660 (5.516) |
| Gastrocnemius sEMG | 0 | 0.263 (0.109) | 0.180 (0.083) | 0.160 (0.106) |
| Gastrocnemius sEMG | 25 | 0.271 (0.069) | 0.244 (0.091) | 0.267 (0.094) |
| Gastrocnemius sEMG | 50 | 0.221 (0.053) | 0.341 (0.094) | 0.218 (0.116) |
| Gastrocnemius sEMG | 75 | 0.224 (0.080) | 0.109 (0.053) | 0.093 (0.072) |
| Gastrocnemius sEMG | 100 | 0.267 (0.109) | 0.191 (0.088) | 0.179 (0.144) |
| Tibialis anterior sEMG | 0 | 0.224 (0.099) | 0.302 (0.054) | 0.499 (0.122) |
| Tibialis anterior sEMG | 25 | 0.147 (0.076) | 0.212 (0.075) | 0.153 (0.058) |
| Tibialis anterior sEMG | 50 | 0.168 (0.108) | 0.104 (0.058) | 0.079 (0.063) |
| Tibialis anterior sEMG | 75 | 0.216 (0.070) | 0.322 (0.098) | 0.354 (0.143) |
| Tibialis anterior sEMG | 100 | 0.225 (0.096) | 0.315 (0.081) | 0.471 (0.100) |

**C. Reporting conventions for waveform-level descriptive summaries**

| **Element** | **Reporting convention applied** |
| --- | --- |
| State ordering | States were ordered by the retained solution label and kept consistent across all panels in the same figure set. |
| Waveform summary | State-specific mean curves were displayed with an accompanying dispersion band when within-state summaries remained sufficiently stable. |
| Axis handling | Gait cycle was displayed on a common 0%–100% x-axis and native domain units were retained on the y-axis. |
| Color logic | One stable color per retained gait state was used across all back-projected domains. |
| Clinical naming | Clinical severity labels were not assigned because the latent-state solution was not externally validated. |
| Interpretive stance | Back-projection was used to support waveform interpretability and not direct controller validation. |

Note. The numerical summaries in Table S5.2 provide compact support for waveform-level interpretation of the retained latent states rather than an exhaustive atlas of every side-aware view. Waveform-level summaries support interpretability of the retained latent states, but they do not, by themselves, validate a neurorobotic controller, decoder, or deployment-ready state estimator.

Acronyms. sEMG = surface electromyography.

**D. State-descriptor labels used for cautious biomechanical interpretation**

| **Retained state** | **Interpretive descriptor** | **Key waveform support** | **Boundary of interpretation** |
| --- | --- | --- | --- |
| **State 1** | **Ankle-limited distal organization** | **Lowest bilateral-mean ankle angle at 50% of the gait cycle and lowest knee angle at 75% among the retained states.** | **Descriptor of waveform organization only; not a severity label, diagnostic subtype, or controller-ready state.** |
| **State 2** | **Intermediate gastrocnemius-centered organization** | **Broadest intermediate latent territory and highest gastrocnemius sEMG activity at 50% of the gait cycle.** | **Descriptor of waveform organization only; push-off relevance is conceptual because no controller or kinetic validation was performed.** |
| **State 3** | **Dorsiflexor/swing-flexion-accentuated organization** | **Highest tibialis anterior sEMG activity at 0%, 75%, and 100% and highest knee angle at 75% of the gait cycle.** | **Descriptor of waveform organization only; not a clinical phenotype or assistance prescription.** |

**Note. These labels were added to make the retained states biomechanically readable while preserving the manuscript claim boundary: the states are representation-level waveform organizations and not clinical labels.**
