# Supplementary material for: Interpretable side-aware kinematic-sEMG gait-state representations relevant to adaptive neurorobotic assistance after stroke: a public-dataset study
Source: Front Neurorobot. 2026 May 25;20:1863916. doi: 10.3389/fnbot.2026.1863916 (PMC13243435; doi:10.3389/fnbot.2026.1863916)
Supplement: Supplementary file 6 [file Data_Sheet_6.docx]

**Supplementary Material 6. Domain-block contribution outputs for the retained solution**

This supplementary file reports the domain-level contribution index for the retained fused side-aware three-state solution. The contribution index was computed directly in standardized waveform space by aggregating the between-state separation fraction across the four side-aware views of each domain and then normalizing the resulting score to the highest-contributing domain. These values are intended as transparent interpretive aids, not as evidence of causal importance.

**Table S6.1. Normalized domain-level contribution index for the retained fused side-aware solution.**

| **Domain** | **Normalized contribution** |
| --- | --- |
| Ankle angle | 1.0 |
| Vastus lateralis sEMG | 0.898 |
| Knee angle | 0.866 |
| Gastrocnemius sEMG | 0.851 |
| Tibialis anterior sEMG | 0.84 |
| Erector spinae sEMG | 0.614 |
| Rectus femoris sEMG | 0.593 |
| Hip angle | 0.588 |
| Semitendinosus sEMG | 0.473 |
| Biceps femoris sEMG | 0.441 |
| Pelvis angle | 0.33 |

*Note. In the retained solution, ankle angle provided the highest normalized contribution index. The remaining contribution pattern remained distributed across distal kinematic, distal myoelectric, proximal, and axial domains, supporting a representation-level interpretation rather than a single-marker interpretation.*
