# Supplementary material for: Interpretable side-aware kinematic-sEMG gait-state representations relevant to adaptive neurorobotic assistance after stroke: a public-dataset study
Source: Front Neurorobot. 2026 May 25;20:1863916. doi: 10.3389/fnbot.2026.1863916 (PMC13243435; doi:10.3389/fnbot.2026.1863916)
Supplement: Supplementary file 7 [file Data_Sheet_7.docx]

**Supplementary Material 7. Subject-level state-assignment matrix and assignment summary**

This supplementary file provides the subject-level matrix for the retained solution. All 50 stroke participants are listed. The seven participants with missing paired lower-limb normalized sEMG were excluded from the strict fused complete-case cohort and therefore do not receive a retained state assignment in Table S7.1. Family PC1 and Family PC2 are the first two coordinates of the retained fused family-level reduced space used for subject-level characterization and low-dimensional documentation.

**Table S7.1. Subject-level inclusion flags, retained state assignments, and leading family coordinates.**

| **Subject ID** | **Included in strict fused complete-case cohort** | **Missing paired lower-limb normalized sEMG** | **Missing paired erector spinae normalized sEMG** | **Retained state** | **Family PC1** | **Family PC2** |
| --- | --- | --- | --- | --- | --- | --- |
| Sub01 | Yes | No | No | 1 | -5.412 | -4.452 |
| Sub02 | Yes | No | No | 3 | 6.568 | -2.109 |
| Sub03 | Yes | No | No | 1 | -5.581 | -0.045 |
| Sub04 | Yes | No | No | 2 | 2.99 | 1.212 |
| Sub05 | Yes | No | No | 1 | -7.068 | 1.745 |
| Sub06 | Yes | No | No | 3 | 5.52 | 0.237 |
| Sub07 | Yes | No | No | 2 | -0.162 | 3.301 |
| Sub08 | No | Yes | Yes |  |  |  |
| Sub09 | Yes | No | No | 3 | 3.927 | 1.331 |
| Sub10 | Yes | No | No | 2 | -2.536 | 3.113 |
| Sub11 | Yes | No | No | 1 | -3.102 | -1.509 |
| Sub12 | Yes | No | No | 3 | 6.772 | -4.026 |
| Sub13 | Yes | No | No | 2 | -0.893 | -1.192 |
| Sub14 | Yes | No | No | 2 | 4.045 | 2.907 |
| Sub15 | Yes | No | No | 2 | 0.687 | -1.5 |
| Sub16 | Yes | No | No | 3 | 7 | -1.807 |
| Sub17 | Yes | No | No | 3 | 4.01 | -0.645 |
| Sub18 | Yes | No | No | 2 | -2.925 | 1.569 |
| Sub19 | Yes | No | No | 2 | -1.728 | 7.592 |
| Sub20 | Yes | No | No | 1 | -4.651 | -0.008 |
| Sub21 | Yes | No | No | 2 | -0.196 | 4.77 |
| Sub22 | Yes | No | No | 2 | 1.269 | 3.109 |
| Sub23 | Yes | No | No | 3 | 3.235 | -3.868 |
| Sub24 | Yes | No | No | 1 | -5.805 | -3.155 |
| Sub25 | Yes | No | No | 1 | -7.674 | 4.086 |
| Sub26 | Yes | No | No | 1 | -3.693 | -2.98 |
| Sub27 | Yes | No | No | 2 | 1.838 | -6.796 |
| Sub28 | Yes | No | No | 1 | -5.248 | -0.331 |
| Sub29 | Yes | No | No | 3 | 2.104 | -3.205 |
| Sub30 | Yes | No | No | 2 | -1.422 | 3.451 |
| Sub31 | Yes | No | No | 1 | -6.177 | -3.177 |
| Sub32 | Yes | No | No | 2 | 0.371 | -0.636 |
| Sub33 | Yes | No | No | 2 | -2.889 | 0.651 |
| Sub34 | Yes | No | No | 1 | -4.595 | -0.09 |
| Sub35 | Yes | No | No | 2 | 0.806 | -4.398 |
| Sub36 | Yes | No | No | 1 | -5.97 | -9.081 |
| Sub37 | No | Yes | No |  |  |  |
| Sub38 | Yes | No | No | 3 | 7.438 | -0.968 |
| Sub39 | Yes | No | No | 3 | 5.904 | 3.233 |
| Sub40 | Yes | No | No | 2 | -3.115 | 3.248 |
| Sub41 | Yes | No | No | 2 | -3.404 | 3.801 |
| Sub42 | Yes | No | No | 2 | -1.212 | 2.656 |
| Sub43 | No | Yes | Yes |  |  |  |
| Sub44 | Yes | No | No | 3 | 8.442 | -0.849 |
| Sub45 | No | Yes | No |  |  |  |
| Sub46 | No | Yes | No |  |  |  |
| Sub47 | No | Yes | Yes |  |  |  |
| Sub48 | No | Yes | Yes |  |  |  |
| Sub49 | Yes | No | No | 3 | 5.987 | 1.415 |
| Sub50 | Yes | No | No | 3 | 6.542 | 3.402 |

**Table S7.2. State-level summary of the retained family coordinates.**

| **State** | **Subjects** | **Family_PC1_mean** | **Family_PC2_mean** | **Family_PC1_sd** | **Family_PC2_sd** |
| --- | --- | --- | --- | --- | --- |
| 1 | 12 | -5.415 | -1.583 | 1.297 | 3.348 |
| 2 | 18 | -0.471 | 1.492 | 2.163 | 3.404 |
| 3 | 13 | 5.65 | -0.605 | 1.825 | 2.455 |

*Note. In the retained solution, State 1 contained 12 subjects, State 2 contained 18 subjects, and State 3 contained 13 subjects after ordering states by the first family-level principal-component centroid from left to right in the reduced space.*

**Table S7.3. Included-versus-excluded comparison on available kinematic waveform summaries**

**All 50 stroke participants had complete paired sagittal kinematic waveforms in the public spreadsheet export. The seven participants excluded from the strict fused complete-case analysis were excluded because paired lower-limb normalized sEMG was incomplete, not because kinematic waveforms were unavailable. The table below compares included and excluded participants using available bilateral-mean kinematic summaries and mean absolute side-difference summaries across the gait cycle. Values are mean (SD). No inferential testing was performed because the excluded group was small (n = 7) and the comparison is intended as a missingness description, not as a powered group analysis.**

| **Available kinematic summary** | **Included complete-case participants (n = 43)** | **Excluded participants (n = 7)** | **Interpretive note** |
| --- | --- | --- | --- |
| **Ankle angle bilateral cycle mean** | **6.328 (4.168)** | **3.467 (4.014)** | **Kinematics available in both groups.** |
| **Knee angle bilateral cycle mean** | **23.538 (7.979)** | **23.005 (5.247)** | **Kinematics available in both groups.** |
| **Hip angle bilateral cycle mean** | **15.035 (8.994)** | **18.718 (8.921)** | **Kinematics available in both groups.** |
| **Pelvis angle bilateral cycle mean** | **5.950 (5.783)** | **8.925 (5.847)** | **Kinematics available in both groups.** |
| **Ankle angle mean absolute side difference** | **6.247 (4.199)** | **6.383 (3.812)** | **Directional state discovery used signed paretic-minus-non-paretic waveforms; this summary reports absolute magnitude only for missingness description.** |
| **Knee angle mean absolute side difference** | **9.000 (5.627)** | **10.111 (4.542)** | **Directional state discovery used signed paretic-minus-non-paretic waveforms; this summary reports absolute magnitude only for missingness description.** |
| **Hip angle mean absolute side difference** | **6.200 (3.658)** | **8.261 (4.519)** | **Directional state discovery used signed paretic-minus-non-paretic waveforms; this summary reports absolute magnitude only for missingness description.** |
| **Pelvis angle mean absolute side difference** | **2.225 (1.782)** | **2.176 (0.767)** | **Directional state discovery used signed paretic-minus-non-paretic waveforms; this summary reports absolute magnitude only for missingness description.** |

**Note. Imputation-based sensitivity analysis was not performed because the missing values were complete bilateral sEMG waveform channels at the public-export layer. Imputing them would have synthesized high-dimensional myoelectric trajectories rather than testing observed public waveforms. The manuscript therefore treats missing paired lower-limb sEMG as a major limitation and uses strict complete-case fused modelling.**
