# Supplementary material for: Interpretable side-aware kinematic-sEMG gait-state representations relevant to adaptive neurorobotic assistance after stroke: a public-dataset study
Source: Front Neurorobot. 2026 May 25;20:1863916. doi: 10.3389/fnbot.2026.1863916 (PMC13243435; doi:10.3389/fnbot.2026.1863916)
Supplement: Supplementary file 8 [file Data_Sheet_8.docx]

**Supplementary Material 8. Reproducibility workflow, software environment, and implementation notes.** This supplementary material documents a transparent implementation path for the workflow. It is written so that another analyst can reproduce the state-representation pipeline from the public repository layer without depending on hidden source structures. The tables distinguish the software environment from the logical execution order so that reproducibility can be documented independently of one specific coding style.

**A. Reproducible software environment**

| **Component** | **Recommended implementation** | **Reproducibility note** |
| --- | --- | --- |
| Input layer | Direct manuscript-level inputs: MAT_normalizedData_AbleBodiedAdults_v06-03-23.xlsx, MAT_normalizedData_PostStrokeAdults_v27-02-23.xlsx, MATdatafiles_description_v1.3_LST.xlsx, README.txt, and ExampleCode_LoadStruct_PlotTimeNormVar_v1.txt | These files are sufficient for the spreadsheet-layer workflow; the raw C3D/PiG archives, time-normalized figure archives, and data descriptor paper remain contextual rather than direct analytic inputs |
| Primary data handling | Participant-level waveform import, workbook-integrity screening, verification of 1001-sample gait-cycle trajectories, and confirmation of side-coded stroke exports | Exact side-aware construction is anchored in the public Pside_/Nside_ spreadsheet layer and the repository descriptor/example code |
| Core numerical tasks | Shared-domain restriction, side-aware view construction, within-cohort pointwise standardization, FPCA, family-level reduction, clustering, repeated 80% subsampling, permutation analysis, and plotting | Candidate screening used Ward hierarchical and K-means across 2-5 states; the retained solution was the fused side-aware K-means three-state solution |
| Suggested open-source implementation | Python 3.x with pandas, numpy, scipy, scikit-learn, matplotlib, and openpyxl | This is one transparent implementation route rather than the only permissible environment |
| Randomness control | Set and archive all random seeds used in clustering, resampling, and permutation procedures | Exact seed values were not enumerated in the archived manuscript materials and should therefore accompany any executable release |
| Output registry | Reduced feature matrices, latent coordinates, state labels, stability summaries, back-projected waveforms, domain-importance tables, and subject-level assignment outputs | All outputs should be versioned and linked to the exact representation family, scaling scheme, and retained solution labels |

**B. Deterministic execution order**

| **Stage** | **Execution note** |
| --- | --- |
| Step 1 | Import MAT_normalizedData_AbleBodiedAdults_v06-03-23.xlsx and MAT_normalizedData_PostStrokeAdults_v27-02-23.xlsx and verify workbook integrity against MATdatafiles_description_v1.3_LST.xlsx, README.txt, and ExampleCode_LoadStruct_PlotTimeNormVar_v1.txt |
| Step 2 | Restrict the signal space to the eleven waveform domains genuinely shared across the public able-bodied and stroke exports |
| Step 3 | Construct the side-aware representation views permitted by the public stroke exports: paretic, non-paretic, bilateral-mean, and side-difference |
| Step 4 | Apply within-cohort pointwise standardization as the primary scaling step and run robust-scaling sensitivity analyses |
| Step 5 | Reduce dimensionality within each domain-view combination to 90% cumulative variance with a cap of three components per block, then assemble the representation families and reduce them to 90% cumulative variance with a cap of eight family components |
| Step 6 | Run Ward hierarchical and K-means clustering across two- to five-state solutions within each representation family and retain the fused side-aware K-means three-state solution |
| Step 7 | Apply repeated 80% subsampling, sensitivity analyses, and explainability procedures to the retained solution; exact repetition counts and the exact surrogate-model specification were not enumerated in the archived manuscript materials |
| Step 8 | Export manuscript-facing outputs, including candidate-screening tables, state assignments, low-dimensional displays, waveform summaries, and domain-contribution tables |

Note. Manuscript-level reproducibility refers to deterministic reconstruction of the representation workflow from the public waveform layer. The archived materials document the exact public inputs, waveform structure, shared-domain restriction, dimensionality caps, candidate clustering screen, and 80% subsampling design. They do not themselves enumerate the exact random seed values, the exact number of resampling or permutation repetitions, or the exact surrogate-model specification used for explainability. Exact regeneration of stochastic state-assignment and explainability outputs therefore requires those implementation metadata to be archived alongside any executable release.

**C. Exact documented specifications and manuscript-level reproducibility boundaries**

**Table S8.3. Exact workflow specifications recoverable from the archived manuscript materials and implementation details not explicitly archived.**

| **Item** | **Specification recoverable from archived materials** | **Manuscript-level status** |
| --- | --- | --- |
| Direct analytic inputs | MAT_normalizedData_AbleBodiedAdults_v06-03-23.xlsx; MAT_normalizedData_PostStrokeAdults_v27-02-23.xlsx; MATdatafiles_description_v1.3_LST.xlsx; README.txt; ExampleCode_LoadStruct_PlotTimeNormVar_v1.txt | Documented |
| Functional object | One participant-level waveform per variable; 1001 gait-cycle samples | Documented |
| Stroke side coding | Pside_/Nside_ waveform exports and side-aware paretic/non-paretic construction | Documented |
| Shared analytic space | Eleven waveform domains shared across cohorts | Documented |
| Within-block reduction | FPCA to 90% cumulative variance with a cap of three components per block | Documented |
| Family reduction | 90% cumulative variance with a cap of eight family components | Documented |
| Candidate clustering screen | Ward hierarchical and K-means clustering across two- to five-state solutions; retained solution = fused side-aware K-means, three states | Documented |
| Stability design | Repeated 80% subsampling with bootstrap ARI summaries | Design documented; exact repetition count not explicitly archived |
| Random seeds | Random seeds were to be recorded for clustering, resampling, and permutation procedures | Exact values not explicitly archived |
| Explainability model and permutations | Transparent surrogate model plus domain-block permutation analysis | Exact surrogate-model class/hyperparameters and exact permutation count not explicitly archived |
| Contextual but non-direct files | Time-normalized figure archives, raw PiG/C3D archives, and the data descriptor paper | Contextual only; not required for spreadsheet-layer reproduction |

**Acronyms.** FPCA = functional principal component analysis; sEMG = surface electromyography.

**Note. The manuscript now distinguishes deterministic reconstruction of the public-export workflow from exact stochastic regeneration of state labels and permutation-style summaries. Exact random seed values, exact resampling counts, and exact permutation counts should accompany any executable code release; where these values were not fully enumerated in the manuscript-level archive, this is stated explicitly rather than implied.**
